# Stabilization of copper nanoparticles onto the double Schiff-base-functionalized ZSM-5 for A^3^ coupling reaction catalysis aimed under mild conditions†

**DOI:** 10.1039/d2ra07700k

**Published:** 2023-02-07

**Authors:** Leila Mohammadi, Mojtaba Hosseinifard, Mohammad Reza Vaezi, Sadegh Rostamnia

**Affiliations:** a Department of Nano Technology and Advanced Materials, Materials and Energy Research Center Karaj Iran l.mohammadi3790@gmail.com m_r_vaezi@merc.ac.ir; b Department of Energy, Materials and Energy Research Center Karaj Iran m.hosseini@merc.ac.ir; c Organic and Nano Group (ONG), Department of Chemistry, Iran University of Science and Technology (IUST) PO BOX 16846-13114 Tehran Iran rostamnia@iust.ac.ir

## Abstract

In this research a highly efficient and heterogeneous catalyst of ZSM-5@APTMS@(*E*)-4-((pyridin-2-ylimino)methyl) benzaldehyde@Cu-NPs was synthesized for upgrading the A^3^ coupling reaction for the synthesis of propargylamines under mild conditions. Rational tuning of the microenvironment of metallic NPs to improve efficiency and reusability in catalytic performances is of significance for scalable applications. Firstly, ZSM-5 was immobilized with APTMS (3-aminopropyltrimethoxysilane) and further modified with (*E*)-4-((pyridin-2-ylimino)methyl)benzaldehyde. Subsequently, the amine-activated zeolite@(*E*)-4-((pyridin-2-ylimino)methyl)benzaldehyde was applied to increase the stabilization of Cu metal nanoparticles. The catalyst was treated with hydrazine to reduce Cu(ii) to Cu(0), which led to active metal sites. The results of catalytic performance in comparison with different parts of catalysis indicate high efficiency due to the stabilization of copper nanoparticles onto the newly synthesized support of MOF modified with nitrogen aromatic groups. The addition of N-rich organic ligand through modification alters the electronic structure of the final composite in favor of the progress of the A^3^-coupling reaction. Moreover, the proposed catalyst showed no reduction in the catalytic performance up to four cycles, and a minor loss of efficiency occurs after the seventh cycle. In addition, the catalyst was effectively recycled up to 7 times without leaching of Cu-NPs.

## Abstract 2

In this research a highly efficient and heterogeneous catalyst of ZSM-5@APTMS@(*E*)-4-((pyridin-2-ylimino)methyl)benzaldehyde@Cu-NPs was synthesized for upgrading the A^3^ coupling reaction for the synthesis of propargylamines under mild conditions.

## Graphical abstract

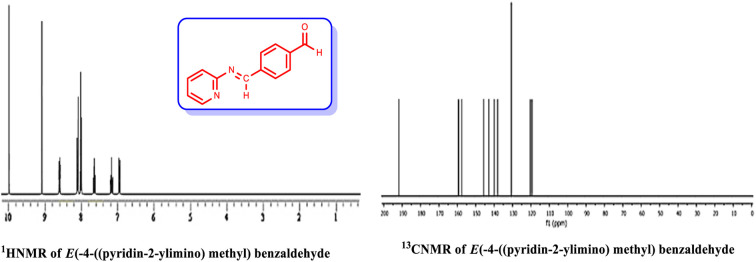

## 1. Introduction

Multi-component responses (MCRs) are focused on synthetic processes involving three or more well-defined reactants to create an item that contains noteworthy fragments of all reactants, exquisitely all atoms.^1–5^ In addition, this class of responses offers a higher level of atomic productivity due to time-saving isolation, as well as the filtration of synthetic intermediates.^6–8^ Lately, a multitude of MCRs, A^3^-type coupling reactions, involving amines and aldehydes by terminal alkynes, directly in a one-pot process, as a robust, valuable, and specific method for a set of complex molecules by noteworthy biological properties through diverse elementary as well as remarkable pioneers has been efficiently developed.^9–13^ Propargylamines which are advantageous platforms, due to economical, easy availability, and adaptability, have been effectively developed in research as compatibility scaffolds for chemical methods and key intermediate drugs to combine biological-active nitrogenous scaffolds including, polyfunctional amino compounds, oxotremorine analogs, β-lactams, as well as pesticides, insecticides, and also medicinal substances for the treatment of Alzheimer's and Parkinson's diseases.^14–17^

Recently, coordinate expansion of terminal alkynes on C–N double bonds arranged from either amines and aldehydes or imines in a one-pot strategy through C–H activated of alkynes through noble transition-metal-catalyzed through homogeneous composites such as Au,^18^ Cu,^19,20^ Ag,^21^ Zn,^22,23^ Ni,^24^ Fe,^25^ Hg,^26^ Co,^27^ Ir,^28^ Ru,^29^ In,^30^ Zr,^31^ Re,^32^ and polyoxovanadate,^33^ as well heterogeneous backed (Ag(i), Au(iii), Cu(ii) and (Cu(i), Ag(i), and Au(i)^34^ were effectively used to catalyze the A^3^ coupling reaction.^35–39^ Copper metal, as one of the most widely utilized metals, has been effectively applied in A^3^-coupling reactions due to its economical availability, and a high potential for reactivity. Nowadays, proficient and versatile solid catalysts such as silicon dioxide, activated charcoal, ZSM-5, Santa Barbara Amorphous-15 (SBA-15),^40^ and also Cu(ii)^41^ complexes by virtue of large surface area caused to effectively adsorb more dynamic location like dynamic metal antecedents and exhibit magnificent catalytic activity are successfully applied to form propargylamine in a one-pot process, which has been more widely preferred to avoid problems such as the accumulation of active sites, toxic metals, and non-recyclable catalysts to minimize risks in biological products.^42–45^

The preparation of organic compounds in green aquatic solvents or solvent-free conditions is a hot topic in today's scientific community.^46,47^ Accomplishments within the field of green chemistry have opened up new prospects for more prominent impacts on the efficiency and performance of chemicals, whereas reducing their adverse effects, also facilitating the safety and benefits of incorporating mild conditions, as well expanding the scope of various organic reactions.^48–50^

ZSM-5 zeolites belong to the pentasil zeolite family, which are crystalline aluminosilicates with periodic arrangements of cages and channels, which have been widely applied as catalysts within the petrochemical industry, adsorbents, and ion exchangers due to their tunable acidity, large surface area, unique shape, high selective stability, as well as uniform micro-porous.^51–54^ Among various zeolites (zeolite Y, beta and mordenite, and ZSM-5), the ZSM-5 catalyst is more valuable for particular aromatic constructions. Medium-pore zeolites like ZSM-5 exhibit increased steric hindrance to reactant molecules within the zeolite pores, resulting in higher aromatics yields and less coke deposition.^55–58^ MFI-type zeolites are generally synthesized in the presence of organic templates, owing to high surface area, interesting channel structure, thermal steadiness, corrosiveness, shape selectivity, and properties of porous ZSM-5; recently, there has been tremendous interest in zeolite-supported heterogeneous catalysts.^59–61^

Here, we have outlined a newly synthesized heterogeneous ZSM-5@APTMS@(terephthalaldehyde/2-aminopyridine)@Cu-NPs catalyst, which has been efficiently used to perform ternary coupling reactions (A^3^-coupling reaction) under mild conditions to successfully promote the preparation of a series of propargylamines with high performance yield.

One reason for the high regeneration performance of the proposed catalyst could be its heightened water resistance as medium-pore zeolites such as ZSM-5 exhibit increased steric hindrance to reactant molecules within the zeolite pores, resulting in higher aromatics yields and less coke deposition, which preserves its structure from destruction by water molecules. Thus, the designed catalysts exhibit superior catalytic performance due to the modulation of the microenvironment of Cu NPs. Also, ZSM-5@APTMS@(*E*)-4-((pyridin-2-ylimino)methyl) benzaldehyde as the catalyst support and Cu nanoparticles as the anchored transition-metal, since the amino group can be firmly attached to the metal nanoparticles. The purpose of ZSM-5 modification was to prepare nitrogen-rich support by providing amino groups to immobilize Cu nanoparticles into it, which led to the high loading of Cu nanoparticles without a noteworthy loss in the leaching of Cu-NPs. Moreover, the proposed catalyst showed excellent recyclability for up to 7 cycles.

## 2. Experimental

### 2.1. Materials and methods

All materials and reagents used in this work were acquired from Merck and Sigma-Aldrich companies and used without further purification.

### 2.2. Synthesis of the *E*(-4-((pyridin-2-ylimino)methyl)benzaldehyde

**Fig. 1:**
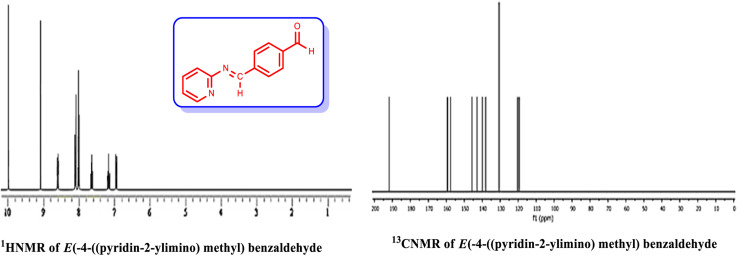
Synthesis of *E*(-4-((pyridin-2-ylimino)methyl)benzaldehyde.

In order to prepare the compound *E*(-4-((pyridin-2-ylimino) methyl) benzaldehyde, the condensation of 2-aminopyridine within terephthalaldehyde was performed based on a report by Gutha *et al.*^62^ In a round bottom flask, (0.2 g) of 2-aminopyridine dissolved in ethanol (25 ml) and (2.81 g) terephthalaldehyde dissolved in methanol (40 ml) were mixed, then acetic acid (3–5 drops) was added dropwise to the flask with stirring at room temperature. Afterward, the reaction mixture was reacted at 65 °C for 2 to 4 h. Then, the reaction mixture was poured over ice (frozen and crushed deionized water) until it was cooled. At this step, yellow sediments were accumulated that were collected by filtration with a Buchner funnel under vacuum. The compound was crystallized using water (Fig. 1).

### 2.3. Synthesis of amine-functionalized ZSM-5 (ZSM-5@APTS, ZSM-5@NH_2_)

A round dry bottom flask was charged with ZSM-5 (a type of MFI zeolite) (1.0 g) and dry toluene (60 ml), then it was dispersed under ultrasonic irradiation for 30 minutes. Afterward, the mixture was completely dispersed and functionalized with APTMS (3-aminopropyltrimethoxysilane) (1.1 ml), diluted in dry toluene (2–3 ml) by adding it dropwise to the flask at room temperature. Afterward, the flask was equipped with a condenser and heated at 80 °C for 24 h. After the reaction was complete, the synthesized amine-functionalized ZSM-5 was filtered, deionized with dry toluene three times, and dried in a vacuum oven at 55 °C for 12 h.

### 2.4. Synthesis of ZSM-5@APTS@*E*(-4-((pyridin-2-ylimino)methyl)benzaldehyde

Zeolite-NH_2_ (0.5 g), prepared in the previous step was dispersed in acetonitrile (CH_3_CN) (25 ml) under stirring for 30 minutes at room temperature. Next, (*E*(-4-((pyridin-2-ylimino(methyl)benzaldehyde) (0.15 g) was completely dissolved in acetonitrile (15 ml) and charged to the mixture and stirred at room temperature for 15 minutes. Then, this mixture was stirred at 75 °C for 24 h. After the reaction time was over. Then, the obtained product was centrifuged, washed with acetonitrile twice, and dried in a vacuum oven at 60 °C for 12 h to provide ZSM-5@APTS@*E*(-4-((pyridin-2-ylimino)methyl)benzaldehyde.

### 2.5. Immobilization of Cu-NPs on ZSM-5@APTS@*E*(-4-((pyridin-2-ylimino)methyl)benzaldehyde

**Scheme 1:**
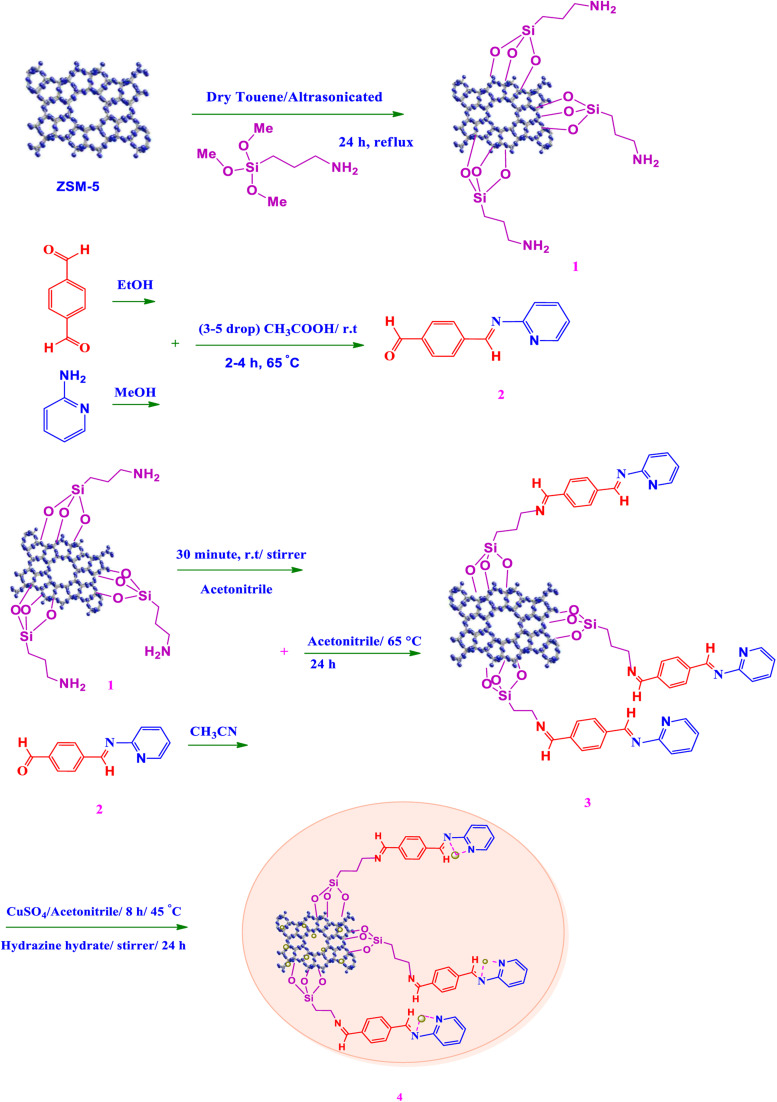
Synthesis of ZSM-5@ZSM-5@APTMS@*E*(4-pyridin-2-(ylimino)methyl)benzaldehyde)/Cu-NPs.

In a round bottom flask, amine-functionalized ZSM-5@(2-aminopyridine/terephthalaldehyde) (0.2 g) in deionized water (50 ml) was added and stirred at room temperature. Afterward, a specified amount of copper(ii) sulfate (CuSO_4_) (0.04 g) was dissolved in deionized water (10 ml) until clear and was added to the flask under stirring at 40–45 °C for 8 h. In order to reduce Cu(ii) to Cu(0), the temperature should be lowered to the ambient temperature. The prepared hydrazine hydrate was completely homogeneous (3 drops of hydrazine hydrate in 3 ml of deionized water) (0.3 ml) and was added to the flask and stirred at room temperature for 24 h, then, Cu(ii) was reduced to Cu (0) with hydrazine hydrate. In the end, the Cu-catalyst was separated easily using a 9000 rpm centrifuge, washed once with acetonitrile, and dried in a vacuum oven at 35 °C for 12 h (Scheme 1).

### 2.6. General procedure for the preparation of the propargylamines

To investigate the catalytic performance of the proposed catalyst, the progress of the A^3^-coupling preparation of propargylamines from the corresponding aldehydes (1 mmol), phenylacetylene (1.1 mmol), and the secondary amines (1 mmol), potassium carbonate as a base (K_2_CO_3_) (2 mmol), solvent water H_2_O (3 ml) and newly synthesized ZSM-5@APTS@(2-aminopyridine/terephthalaldehyde)@Cu-NPs (0.025 g) catalyst were added. The optimum conditions were monitored (Table 2). Purification of the synthesized propargylamines was performed by plate-chromatography. The chemical structure of the synthesized propargylamines was probed by ^1^H NMR and ^13^C NMR (ESI) (Fig. S1†).

## 3. Results and discussion

### 3.1. Catalyst preparation

**Fig. 2:**
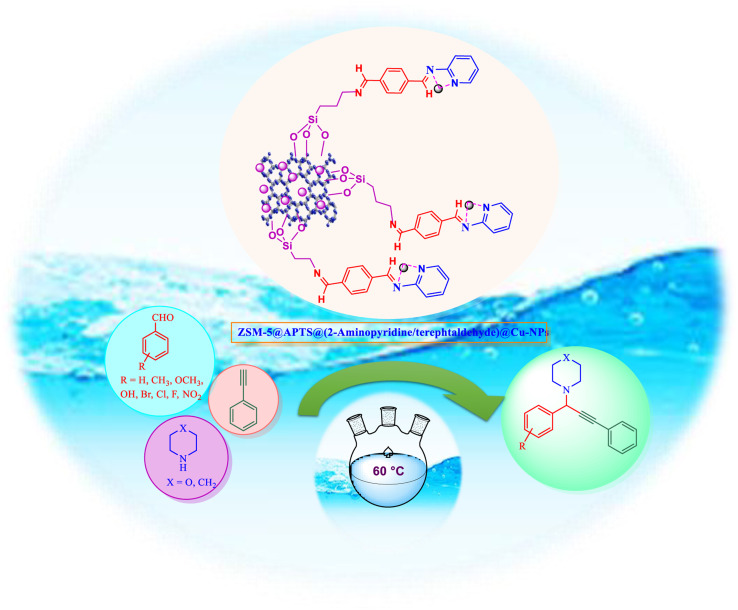
Catalytic performance for the A^3^-coupling reaction.

Following our previous attempts to develop facile and sustainable methodologies for developing various organic reactions,^63–67^ in this report, we introduce a highly efficient and recyclable copper-based catalyst for the promotion of the A^3^-coupling reaction for the preparation of the medicinally important propargylamine derivatives from an aldehyde, an amine, and a terminal alkyne as the starting materials. First, the ZSM-5 was modified with APTMS (3-aminopropyltrimethoxysilane) and further functionalized with (4-pyridine-2-(ylimino)methyl)benzaldehyde). Subsequently, the amine-activated zeolite@(4-pyridin-2-(ylimino)methyl)benzaldehyde) was applied to increase the stabilization of Cu metal nanoparticles. The catalyst was reduced by hydrazine to reduce Cu(ii) to Cu(0) which led to active metal sites (Scheme 1). Finally, the manufactured ZSM-5@APTMS@(4-pyridin-2-(ylimino)methyl)benzaldehyde)/Cu-NPs was employed as a catalyst for the A^3^-coupling preparation of a series of propargylamines (Fig. 2).

### 3.2. Spectroscopic characterization of ZSM-5@APTS@terephthalaldehyde/2-aminopyridine@Cu-NPs catalyst

#### 3.2.1 FT-IR spectroscopy

**Fig. 3:**
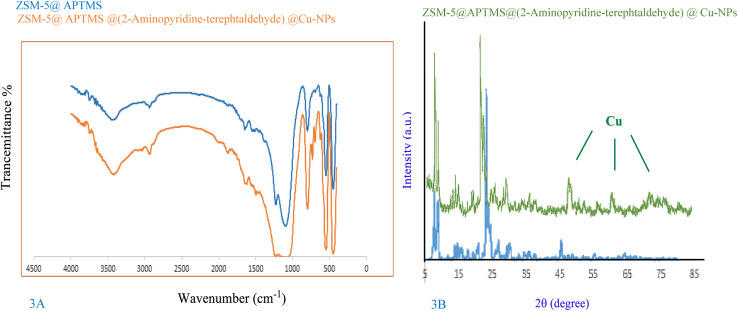
(A) Infrared spectrum (FT-IR) analysis of ZSM-5@ATPS and ZSM-5@APTMS@(2-aminopyridine-terephthalaldehyde)@Cu-NPs, (B) XRD pattern of ZSM-5@APTMS@(2-aminopyridine/terephthalaldehyde)@Cu-NPs.

The FT-IR spectra of ZSM-5@APTMS and the final synthesized catalyst ZSM-5@APTMS@(2-aminopyridine-terephthalaldehyde)@Cu-NPs are shown in Fig. 3A. As indicated in the spectrum, the absorption bands at 3396 cm^−1^, 3429 cm^−1^ are related to O–H extending vibration. It manifests a peak at 3350–3650 cm^−1^, associated with the stretching vibrations of the hydroxyl group. The symmetric and asymmetric stretching vibrational peaks became visible at 791 cm^−1^ and 1076 cm^−1^, which are attributed to the vibrations of the silanol group Si–O–Si. Distinctive bands at 2297 cm^−1^ and 2928 cm^−1^ are related to C–H, and also a characteristic absorption peak at 2939 cm^−1^ corresponds to CH_2_ stretching. Peaks at 1589 cm^−1^, and 1627 cm^−1^ are attributed to (C=C) and the bands corresponding at 1156 cm^−1^ and 1155 cm^−1^ are assumed to be of (C–O) groups, which have distinct functions in the structures. The presence of an asymmetric stretching vibration band around 1225 cm^−1^ confirms the structure of zeolite in the final synthesized catalyst, as well as the bands in the regions of 1100 cm^−1^ and 1400 cm^−1^ are associated with internal tetrahedral asymmetric, stretching vibrations and bending vibration, individually. A band at 1400 cm^−1^ corresponds to the bending of the N–H group. The peak at 3439 cm^−1^ illustrates branching related to the stretching mode of NH_2_ groups, which was modified successfully. Thus, there is strong evidence for the functionalization of ZSM-5. The peak emerging at 1532 cm^−1^ is associated with the stretching vibration of C–N in amine in the ZSM-5@APTMS compound. These outcomes confirm the fruitful fixation of APTMS on the surface of the ZSM-5 substrate. Other characteristic peaks of various parts of the composite overlapped, and other characterization methods were used to prove the formation of the catalyst (Fig. 3A).

#### 3.2.2 X-ray diffraction (XRD) patterns

**Fig. 4:**
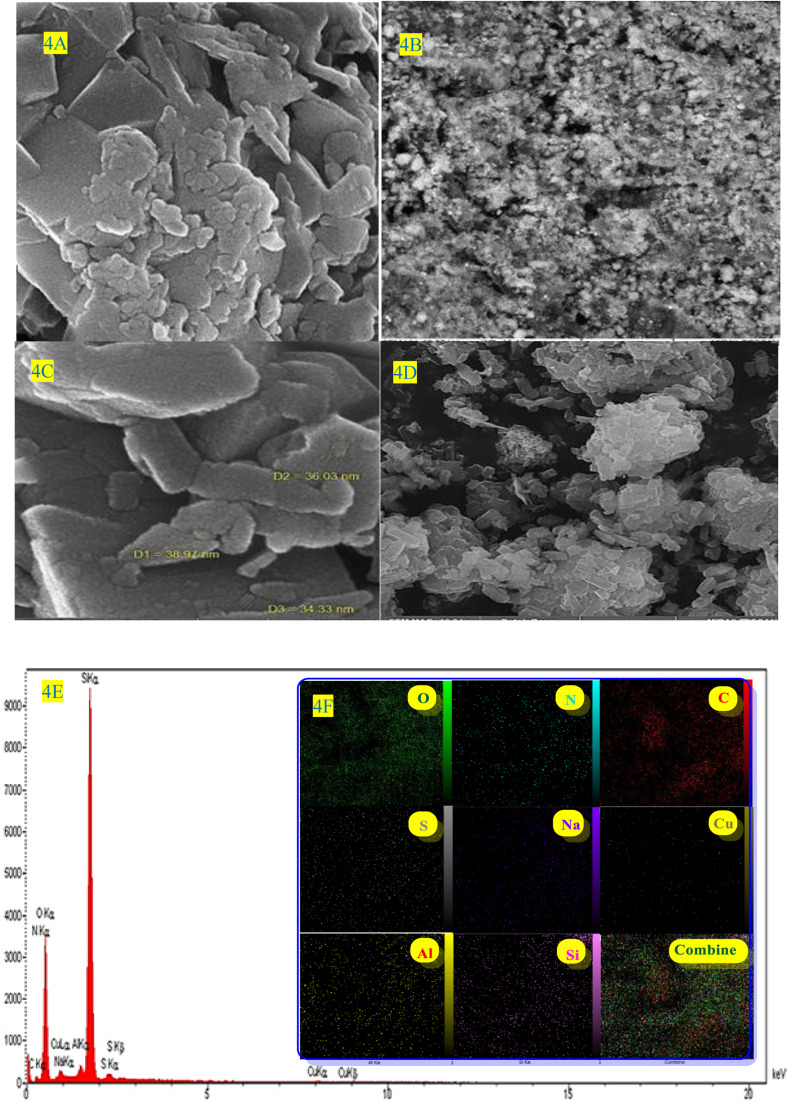
(A–D) the SEM image, (E) EDS, and (F) the mapping spectra of ZSM-5@ATPS@(2-aminopyridine-terephthalaldehyde)@Cu-NPs.

The crystalline structure of ZSM-5@APTMS@(2-aminopyridine-terephthalaldehyde)@Cu-NPs was investigated using the XRD technique (Fig. 4B).

In the XRD pattern of the composition of ZSM-5@APTMS@(2-aminopyridine-terephthalaldehyde) with Cu-NPs, new peaks appeared, which corresponded to the standard Bragg reflections (111), (200), (220) of the Cu-NPs, in accordance with the JCPDS card (No. 71-4610),^68,69^ proving their successful formation.

**Fig. 5:**
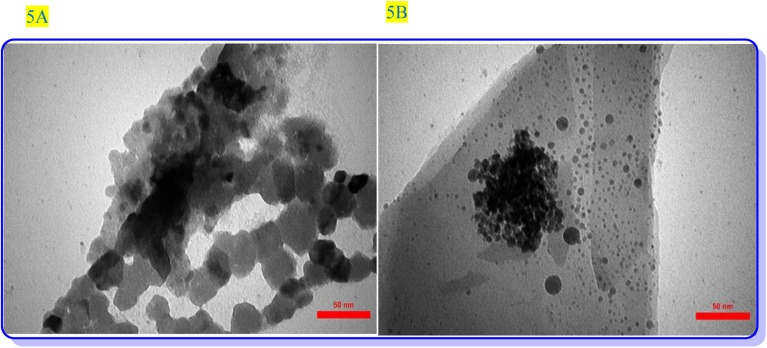
(A and B) The transmission electron microscope (TEM) images of ZSM-5@APTMS@(2-aminopyridine/terephthalaldehyde)@Cu-NPs.

In this context, we used hydrazine hydrate as a reductant, which results in the formation of bigger NPs compared to other reductants such as NaBH_4_, hoping to form Cu-NPs over the surface of the modified ZSM-5. The TEM image exhibits the presence of well-dispersed Cu-NPs over the surface of ZSM-5 (Fig. 5A and B). SEM, EDS, and elemental mapping analysis were used to investigate the elemental composition and spatial distribution of elements in the final composite (Fig. 4A–F, and inset images). SEM-EDS analysis confirmed the presence of Al, S, Si, Na, O, C, N, and Cu in ZSM-5@APTMS@(2-aminopyridine/terephthalaldehyde)@Cu-NPs with 7.42 wt% loading of copper NPs and SEM-elemental mapping exhibits uniform elemental dispersion in the sample matrix, proving its successful formation (Fig. 4A–D).

The morphology and surface structure of ZSM-5@APTMS@(2-aminopyridine/terephthalaldehyde)@Cu-NPs were investigated by SEM and TEM techniques (Fig. 5A and B, respectively). According to Fig. 4A–D, the SEM image shows the nanoparticles with a diameter starting at approximately 34.33–38.97 nm.

Elemental composition and spatial elemental distribution of ZSM-5@APTMS@(2-aminopyridine/terephthalaldehyde)@Cu-NPs were investigated by EDS and mapping techniques (Fig. 4E). According to the EDS data, the atomic ratio of Si/Al is equal to 39, which confirmed the successful immobilization of copper (Cu) nanoparticles in the newly synthesized ZSM-5@APTMS@(2-aminopyridine/terephthalaldehyde)@Cu-NPs catalyst. ZSM-5 catalyst with a high silicon-to-aluminum ratio (Si/Al > 10), indicates an increase in the crystal size, as well as an increase in the surface area, which improves the physicochemical properties of the catalyst.

The transmission electron microscope (TEM) images of the newly synthesized catalyst ZSM-5@APTMS@(2-aminopyridine/terephthalaldehyde)@Cu-NPs illustrate hierarchies of the modified ZSM-5 nanocrystals with intra- and inter-crystalline porous structures (Fig. 5). Low-magnification TEM images confirmed that ZSM-5-modified zeolite@Cu had uniform crystallites of sizes 50 and 100 nm, which is in perfect agreement with the obtained SEM images. Notably, bright spots were observed relative to intra-crystalline mesoporous, many brighter areas correspond to the presented mesoporous and are recognizable.

## 4. Application. Catalytic performance

After careful characterization of the synthesized ZSM-5/APTMS/(4-pyridine-2-(ylimino)methyl)benzaldehyde)/Cu-NPs, we evaluated their catalytic performance for the A^3^-coupling preparation of a series of propargylamine products. To this end, we chose the reaction of benzaldehyde, piperidine, and phenylacetylene as the model reaction. We also examined the effects of the reaction time and temperature, the type of solvent, and the catalyst dosage on the progress of the reaction, and the results are depicted in Table 1. To find the best solvent, the progress of the A^3^-coupling reaction of the model reaction in the presence of the proposed catalyst in various solvents, including water, toluene, DMSO, DMF, CH_2_Cl_2_, MeCN, a mixture of EtOH and H_2_O, EtOH, tetrahydrofuran, and solvent-free conditions were monitored. Despite the remarkable performance of our proposed method in solvent-free conditions, further studies were carried out with H_2_O as the solvent due to its green nature and ease of employment. Studies on the effect of the temperature on the progress of the reaction showed that the best results were obtained at 60 °C, and a further increase in the temperature did not increase the yield of the reaction. We monitored the progress of the reaction by the thin-layer chromatography technique, and we observed that at 60 °C in H_2_O, the reaction reached equilibrium after 120 min. The ideal amount of the catalyst was obtained by tracking the progress of the reaction at various catalyst dosages. The results of this study indicated that 25 mg of the proposed catalyst was sufficient for the optimal progress of the A^3^-coupling reaction. So, based on these studies, 25 mg of ZSM-5/APTMS/(4-pyridine-2-(ylimino)methyl) benzaldehyde)/Cu-NPs catalyst at 60 °C in 3 ml H_2_O as green solvent and base (K_2_CO_3_) (2 mmol) after 120 min gave the highest product yields. In addition, the reaction processes with different types of derivatives aldehydes (with electron-donating and electron-withdrawing groups) and terminal alkynes with different second-type amines were studied (Table 1).

**Table 1:** The results of the optimization studies on the A^3^-coupling preparation of propargylamines in the presence of ZSM-5@APTMS@(2-aminopyridine/terephthalaldehyde)@Cu-NPs^a^

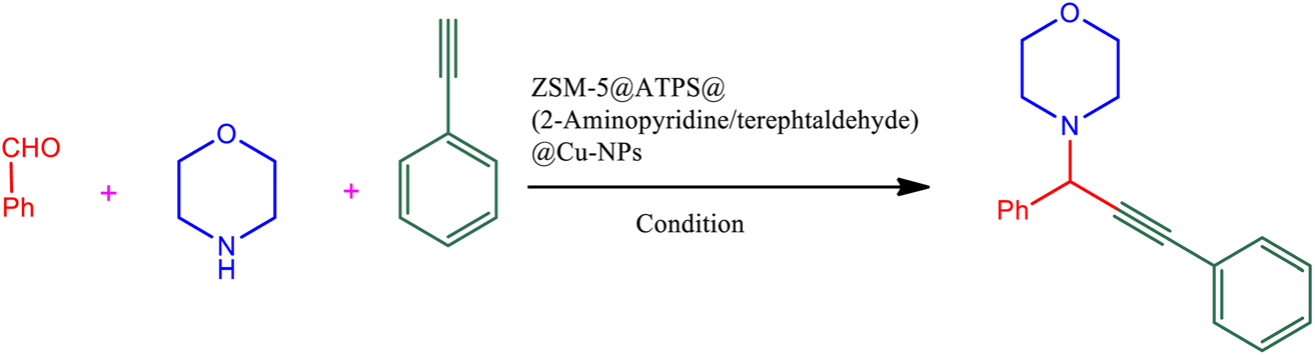						
Entrance	Catalyst (mg)	Solvent	Base	*T* (°C)	Time (min)	Yield (%)
1	—	H_2_O	NaOH	95	250	8
2	5	H_2_O	KOH	95	240	35
3	10	H_2_O	Mg(OH)_2_	90	240	55
4	15	H_2_O	Ca(OH)_2_	85	200	65
5	18	H_2_O	K_2_CO_3_	80	120	70
6	20	H_2_O	K_2_CO_3_	80	120	80
7	25	H_2_O	K_2_CO_3_	75	120	96
8	25	H_2_O	K_2_CO_3_	70	120	95
9	25	H_2_O	K_2_CO_3_	65	120	92
10	25	H_2_O	K_2_CO_3_	60	120	98
11	30	H_2_O	K_2_CO_3_	60	120	92
12	25	—	K_2_CO_3_	100	300	94
13	25	H_2_O	K_2_CO_3_	—	300	10
14	25	H_2_O	—	90	180	73
15	25	PhCH_3_	K_2_CO_3_	100	180	60
16	25	DMSO	K_2_CO_3_	100	200	62
17	25	DMF	K_2_CO_3_	100	200	65
18	25	CH_2_Cl_2_	K_2_CO_3_	45	200	30
19	25	MeCN	K_2_CO_3_	75	200	30
20	25	EtOH/H_2_O	K_2_CO_3_	—	200	60
21	25	EtOH	K_2_CO_3_	78	200	80
22	25	THF	K_2_CO_3_	66	150	35

a Reaction conditions: 1 mmol of morpholine, 1 mmol of benzaldehyde, 1.1 mmol of phenylacetylene, H_2_O (3 ml), K_2_CO_3_ (2 mmol), and catalyst (25 mg).

After obtaining the optimum condition, we tested the generality of our proposed method by synthesizing different propargylamines from various precursors. In this context, diverse aldehydes and amines reacted with the phenylacetylene in the presence of the proposed catalyst under the optimum conditions. Table 2 summarizes the outcomes of this study. As this table indicates, the reaction proceeds in excellent yields in the presence of aromatic aldehydes containing electron donating or withdrawing groups in the aromatic ring's *ortho* or *para* positions. However, employing an aliphatic aldehyde resulted in lower yields. We used three different secondary amines in this reaction, and as shown in Table 2, the change in the amine did not affect the yield of the reaction. These results indicate the excellent performance of the ZSM-5/APTMS/(4-pyridine-2-(ylimino)methyl)benzaldehyde)/Cu-NPs as a catalyst for the promotion of the A^3^-coupling preparation of the propargylamines.

**Table 2:** Preparation of various propargylamines under optimum conditions^a^

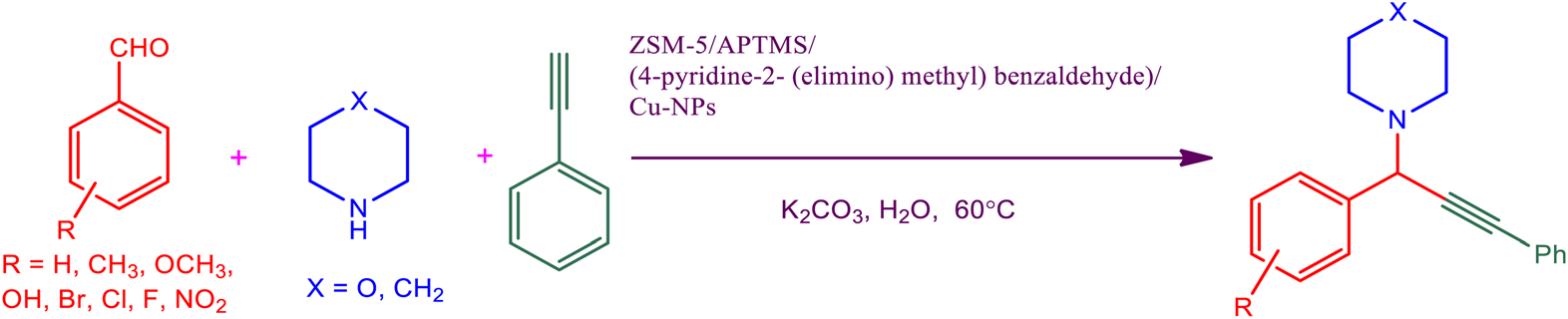					
Entrance	Aldehyde	Amine	Product	Time (min)	Yield (%)
1	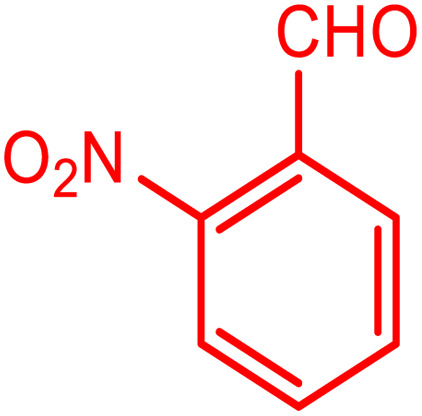	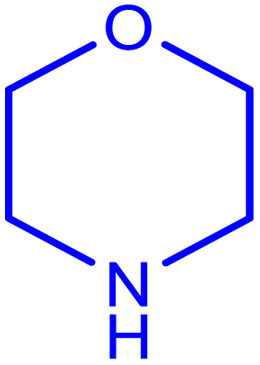	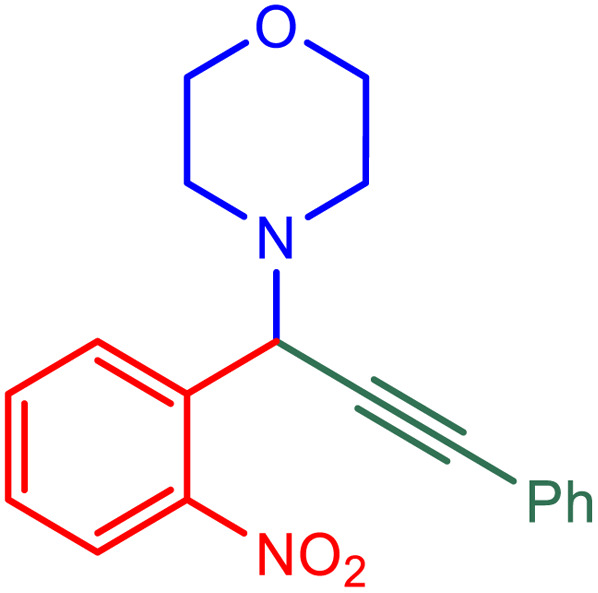	120	99
2	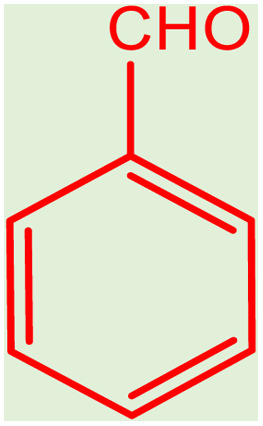	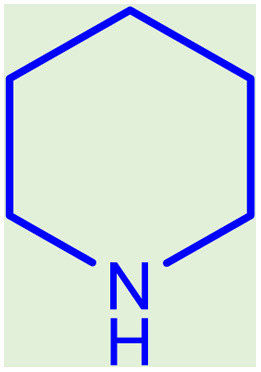	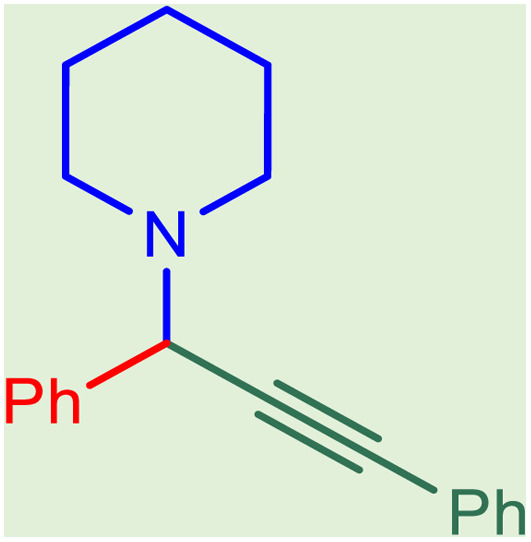	120	98
3	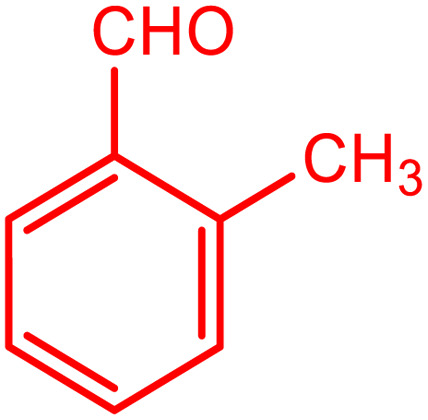	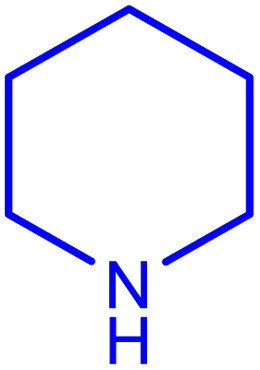	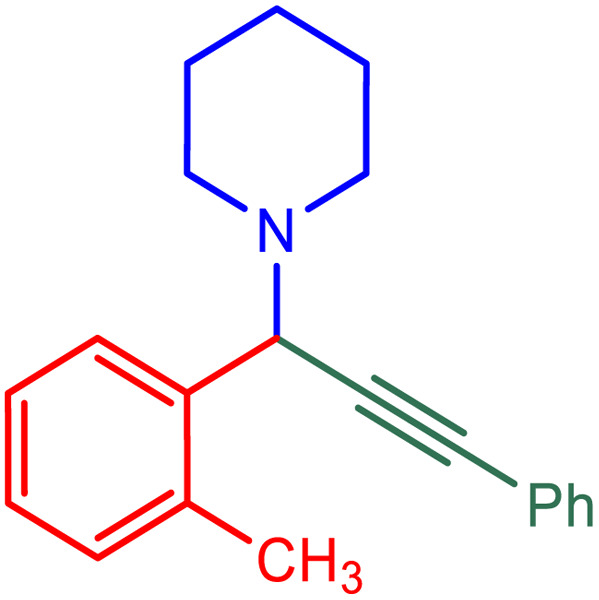	120	91
4	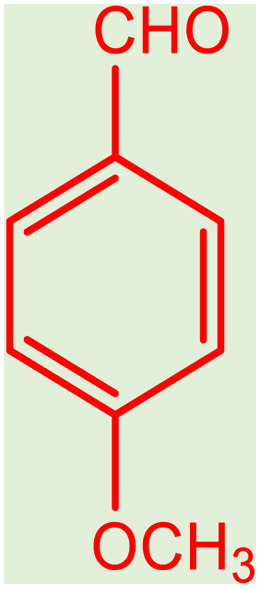	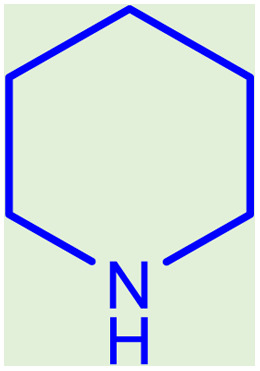	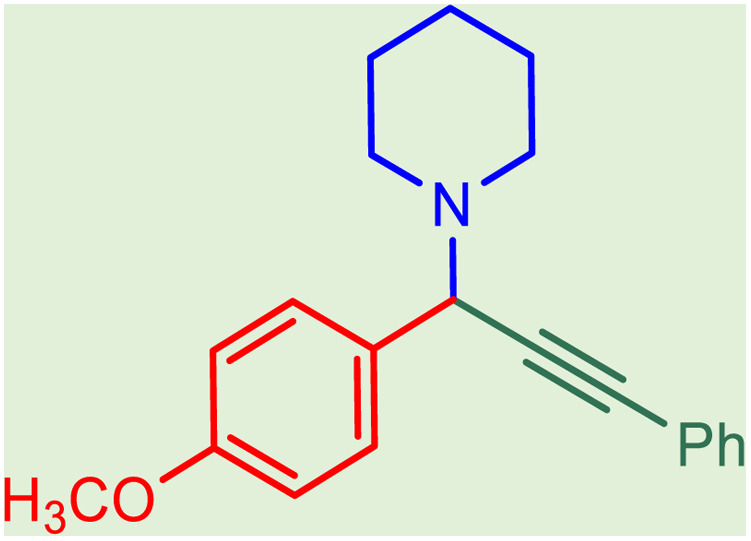	120	90
5	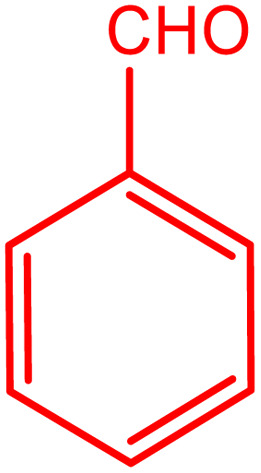	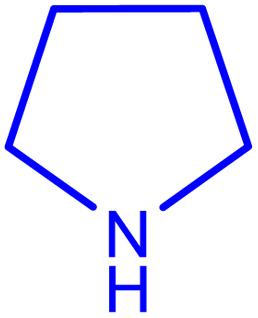	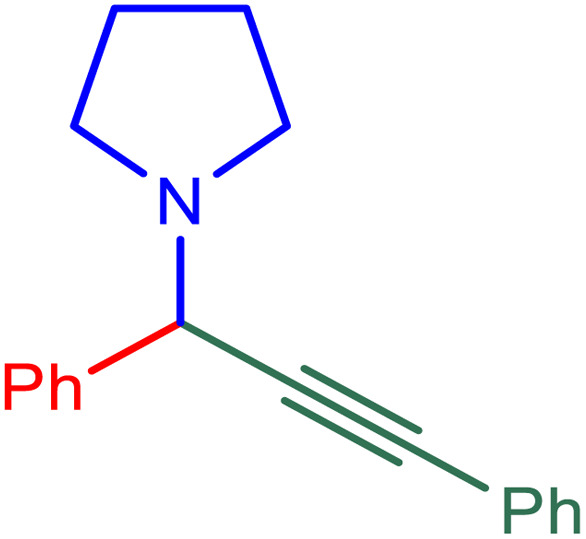	120	93
6	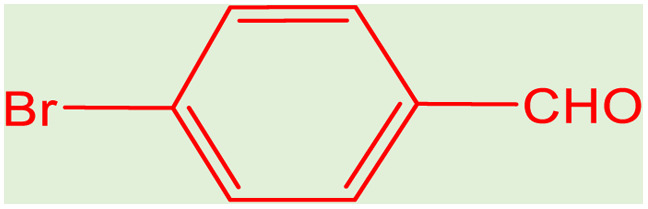	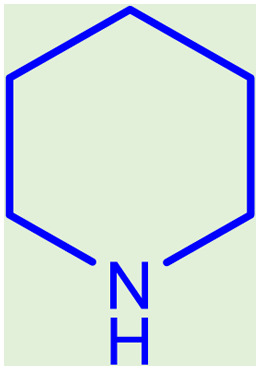	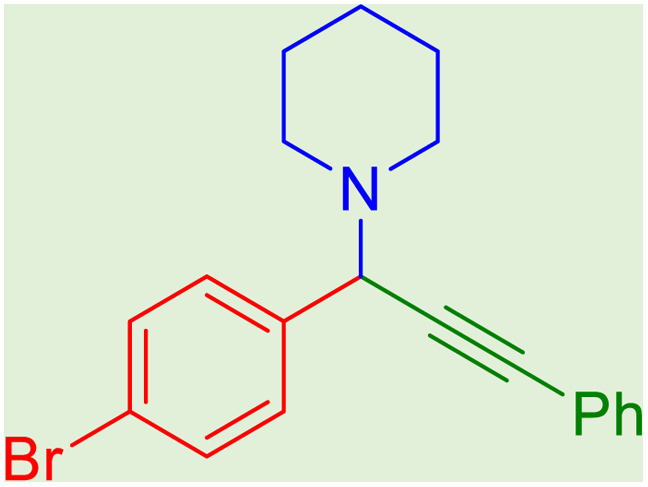	120	95
7	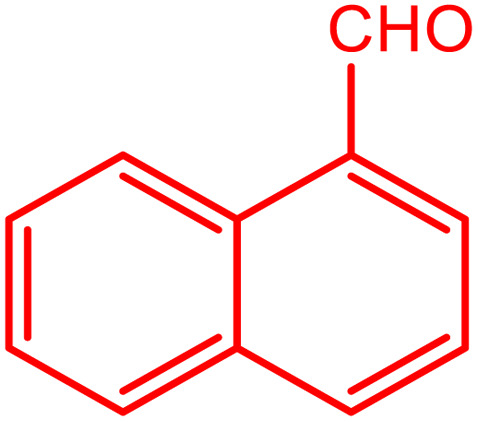	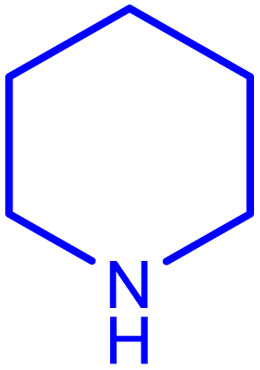	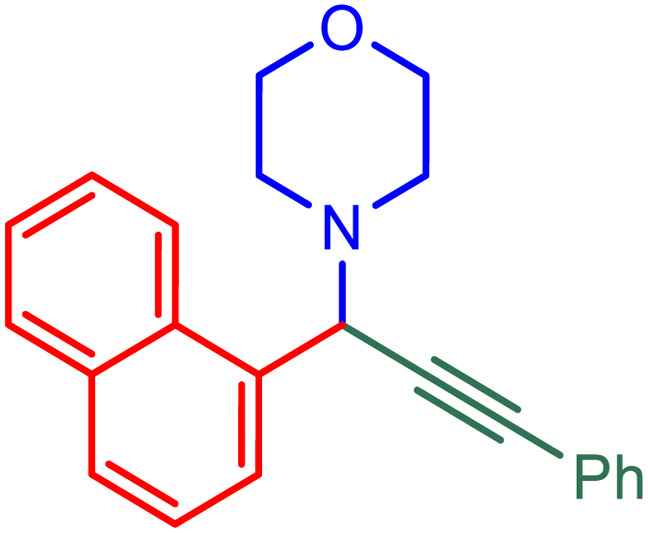	120	92
8	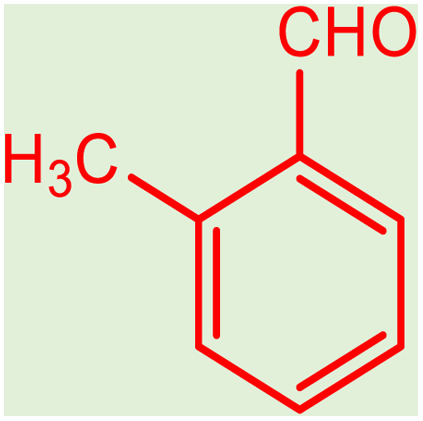	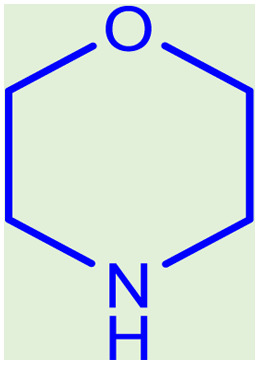	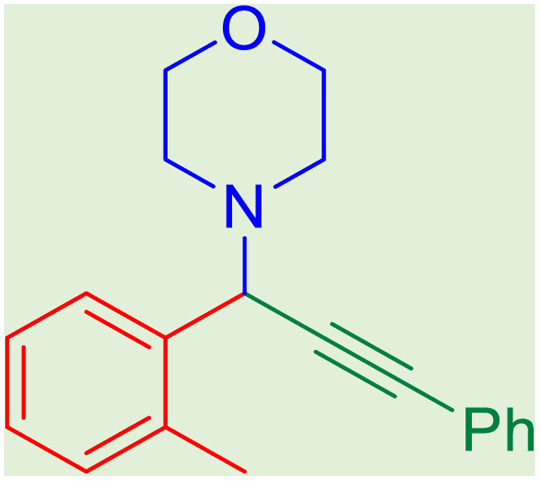	120	91
9	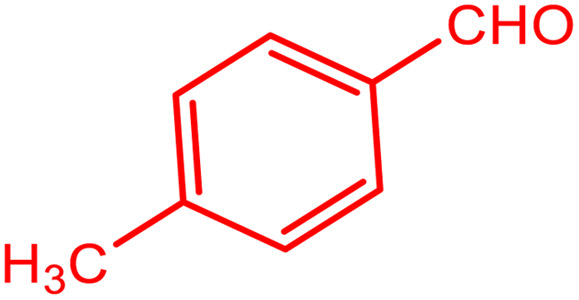	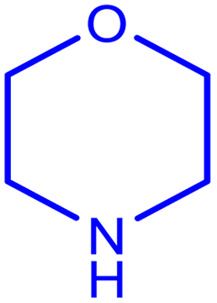	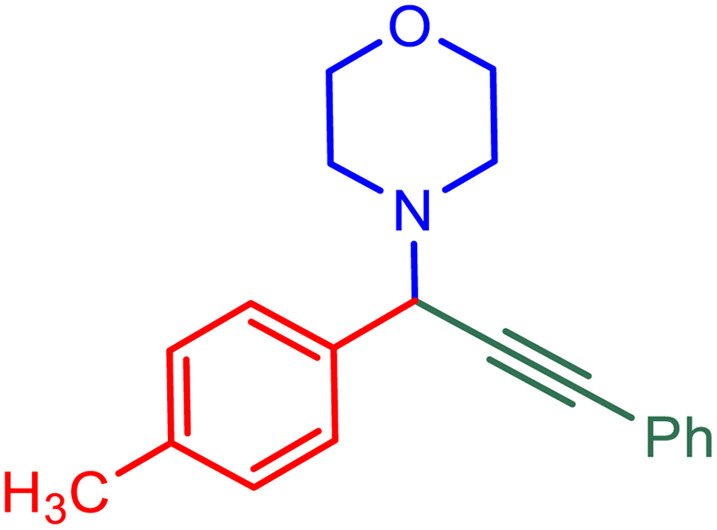	120	92
10	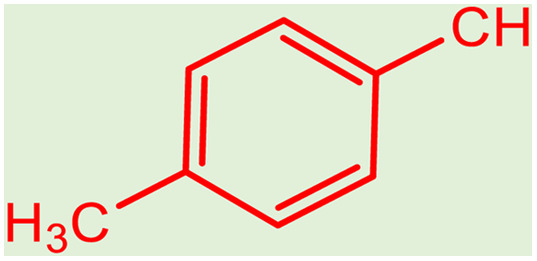	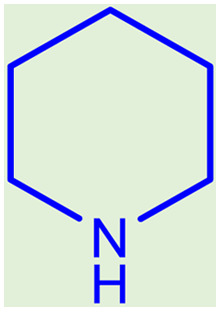	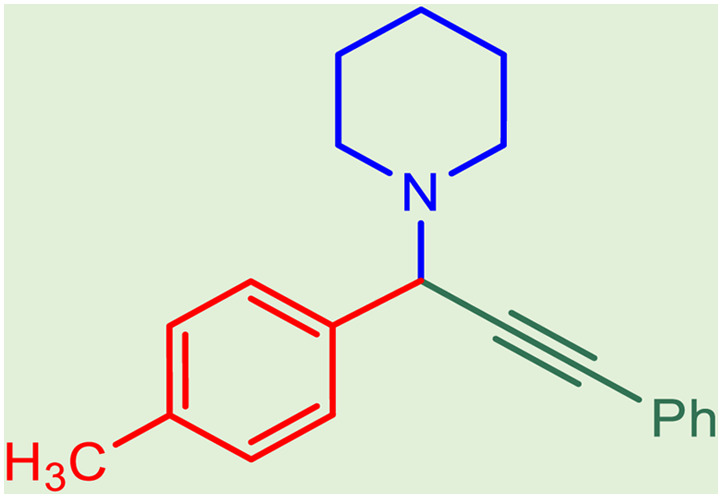	120	94
11	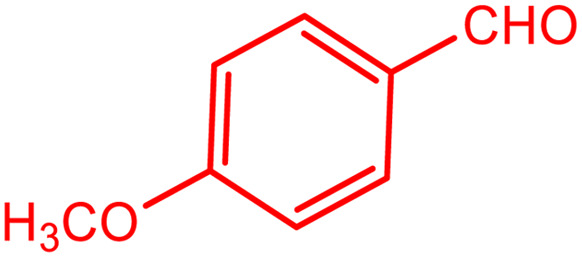	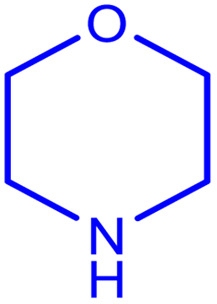	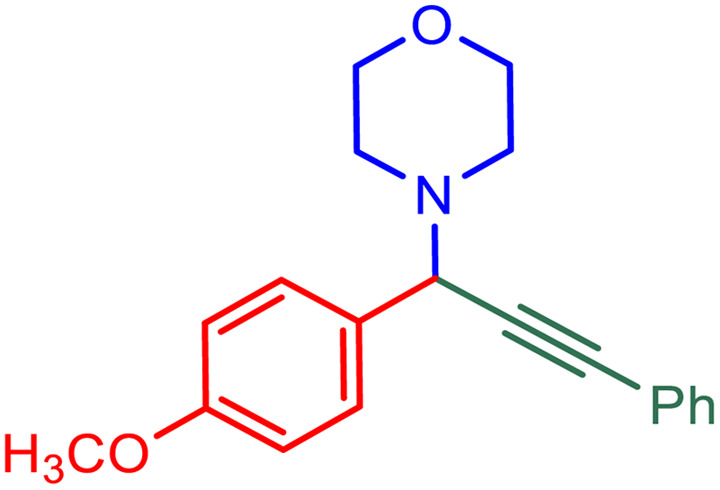	120	90
12	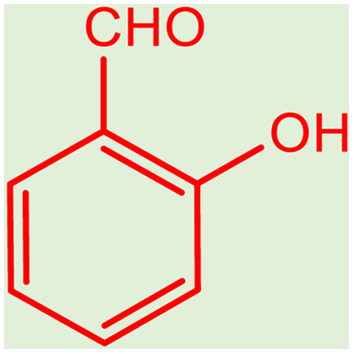	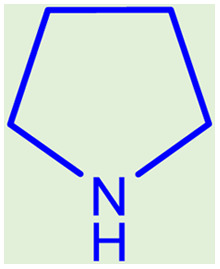	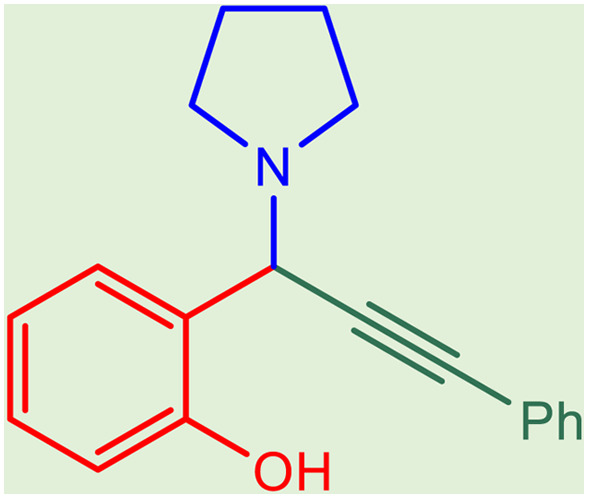	120	95
13	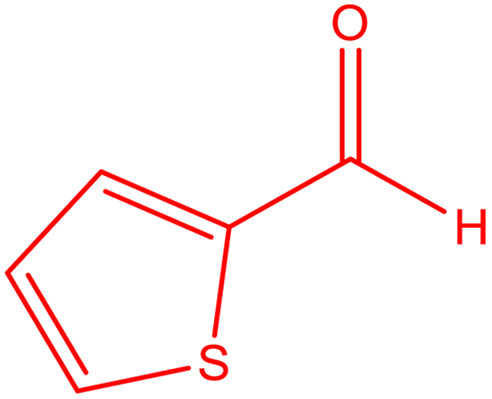	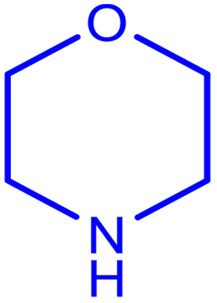	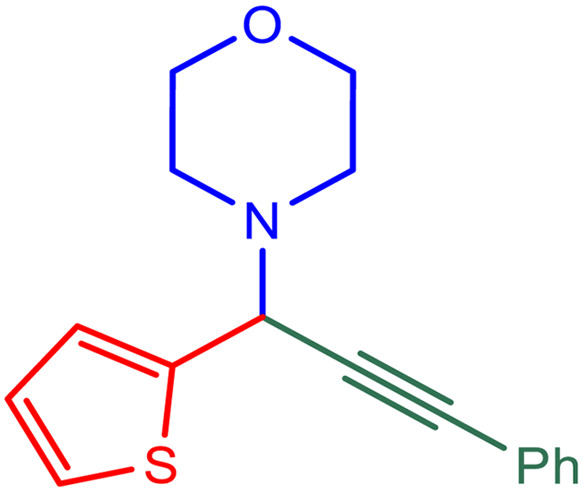	120	93
14	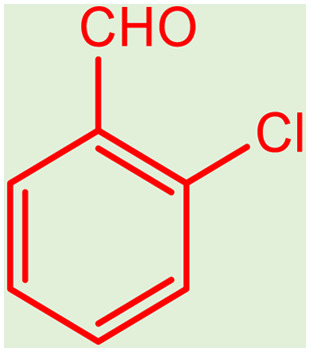	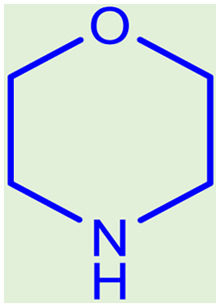	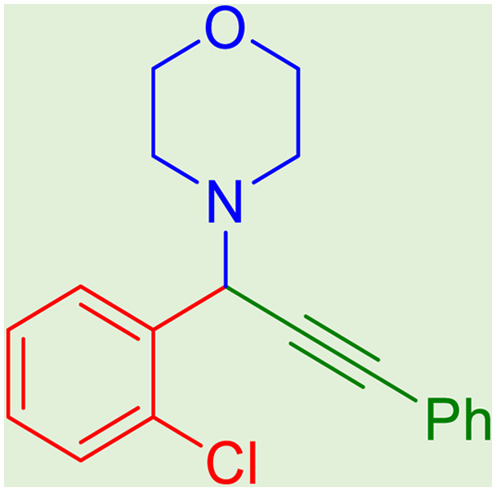	120	92
15	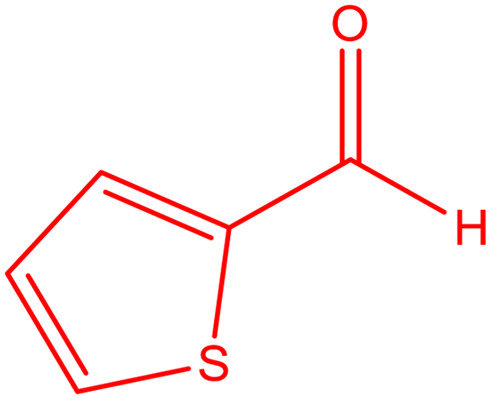	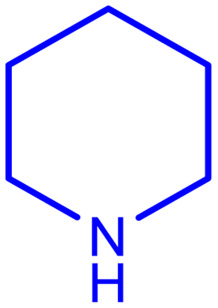	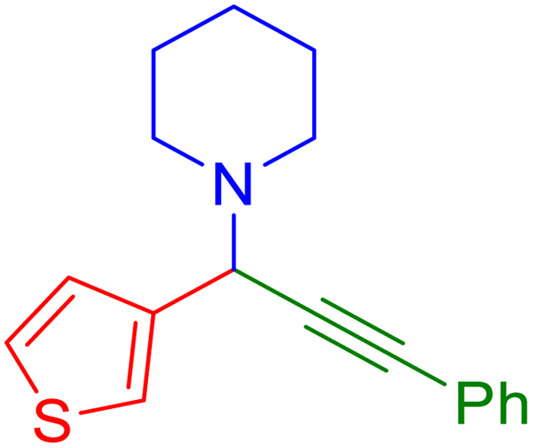	120	94
16	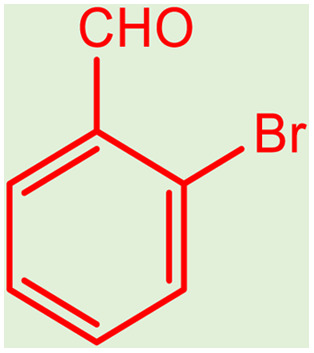	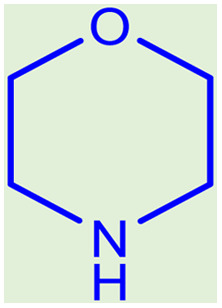	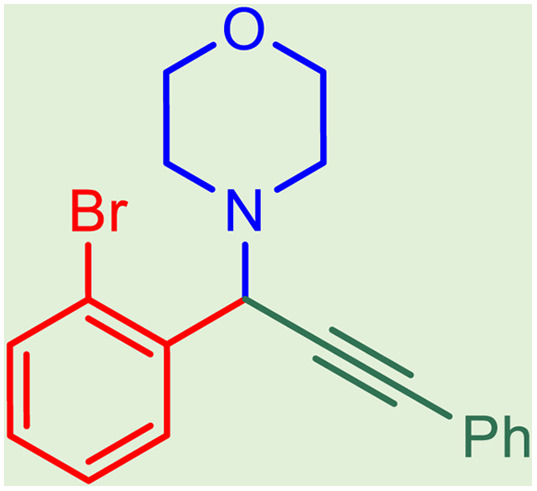	120	93
17	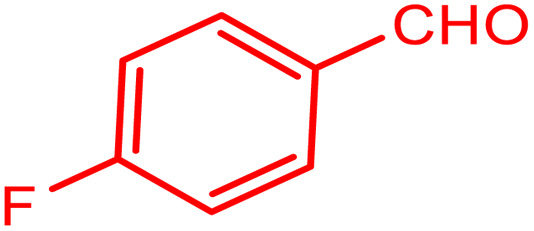	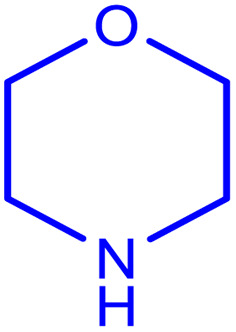	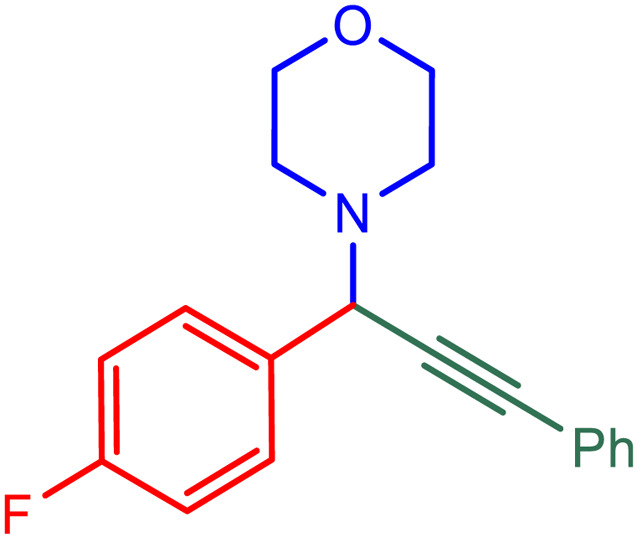	120	95
18	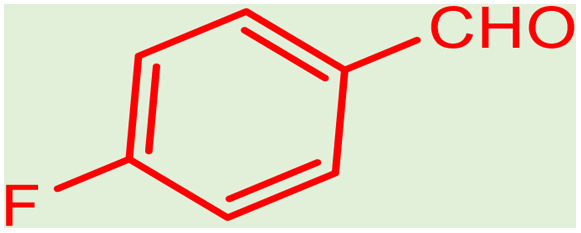	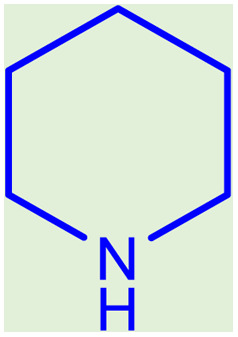	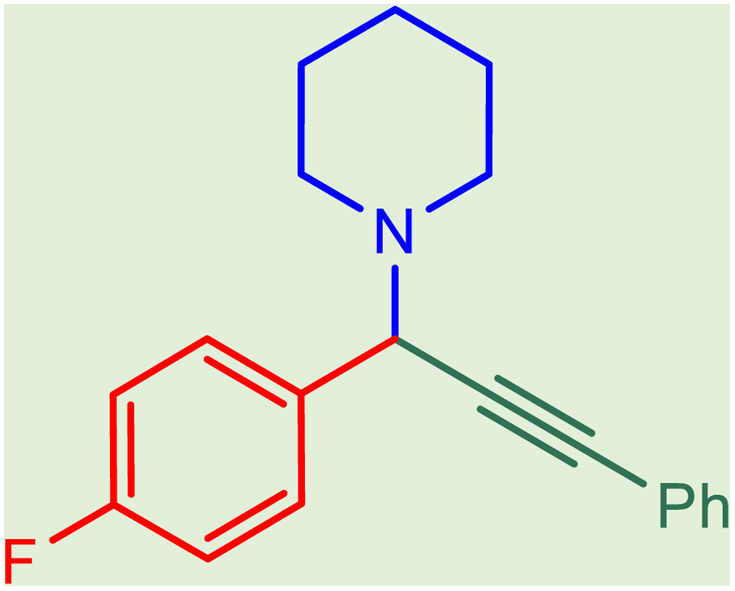	120	96
19	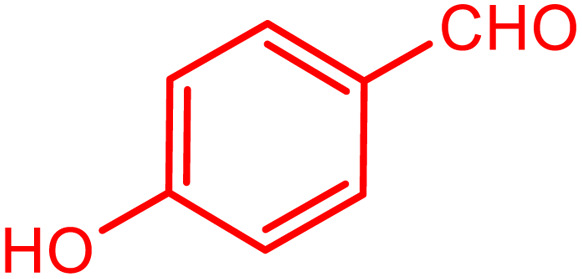	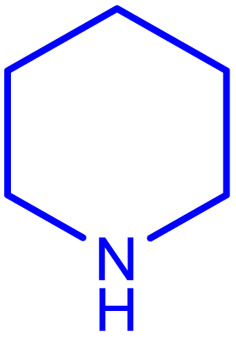	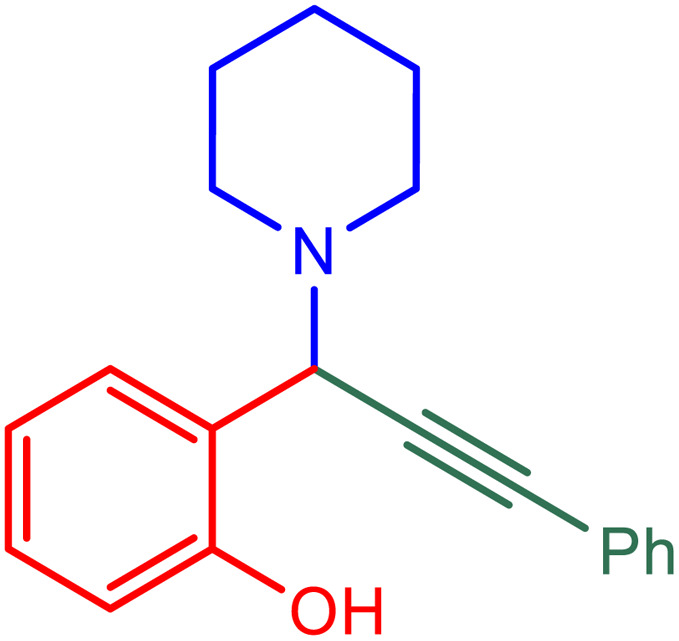	120	97
20	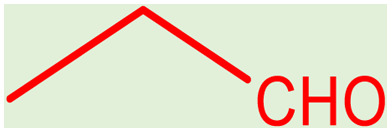	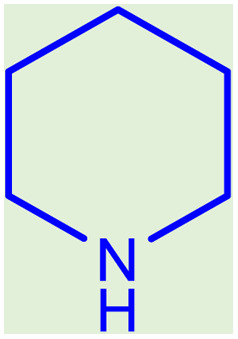	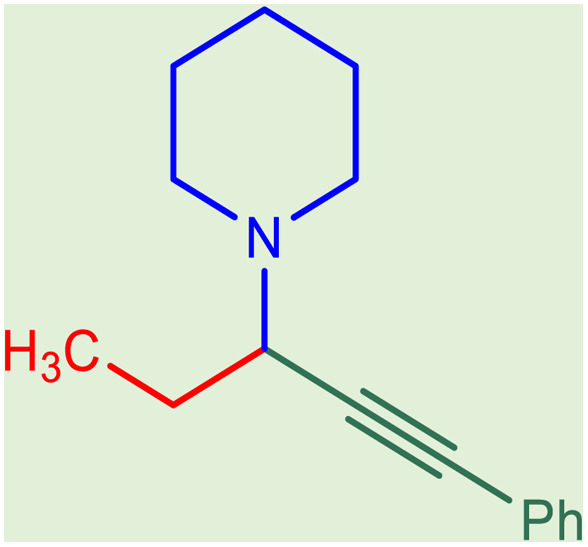	120	85

a Reaction conditions: 1 mmol of morpholine, 1 mmol of benzaldehyde, and 1.1 mmol of phenylacetylene, H_2_O (3 ml), K_2_CO_3_ (2 mmol), and catalyst (25 mg).

## 5. The proposed mechanism

**Fig. 6:**
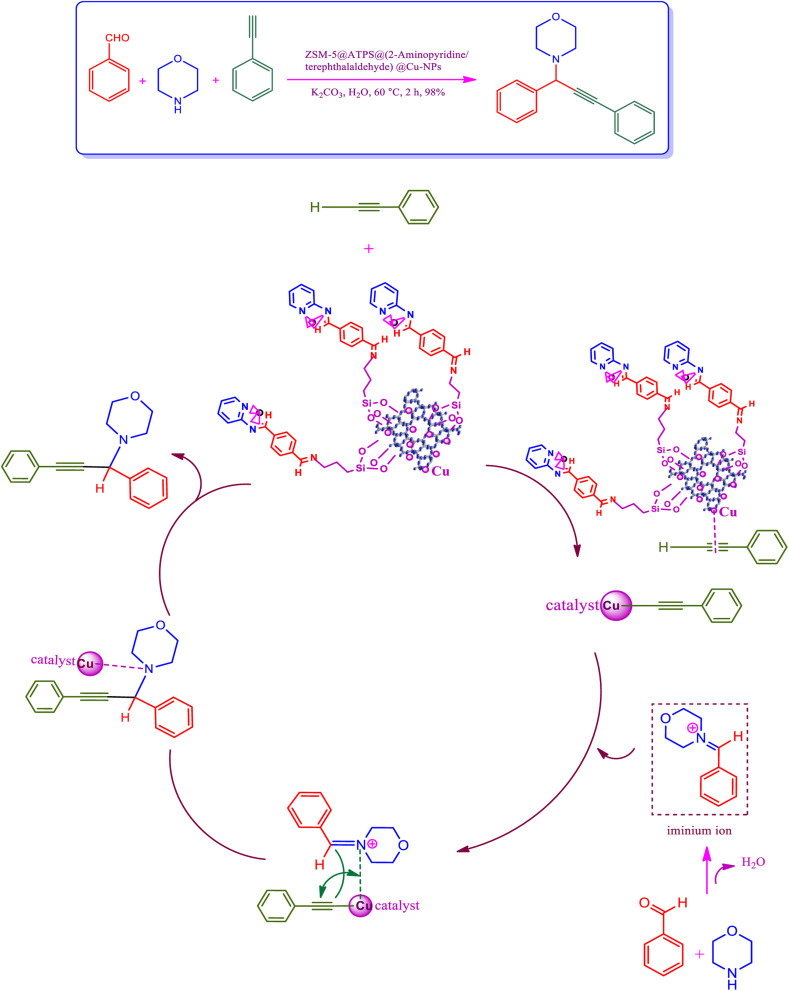
The proposed mechanism to provide propargylamines in the presence of ZSM-5/APTMS/(4-pyridine-2-(ylimino)methyl)benzaldehyde)/Cu-NPs.

The plausible mechanism was performed through the C–H activation of alkyne by the efficient newly synthesized copper-ZSM-5 catalyst to establish copper acetylide, which was performed to develop A^3^ coupling reactions. The first reaction was expected to activate the C–H bond through copper nanoparticles to develop a copper acetylide intermediate.^70^ Following, the prepared copper acetylide intermediate was reacted with the immonium ion, which was provided *via* nucleophilic attack of the secondary amine on the electrophilic carbon of the aldehyde group with the elimination of a water molecule, in order to provide the formation-desired propargylamine. ZSM-5/APTMS/(*E*)-4-((pyridin-2-ylimino) methyl) benzaldehyde@Cu-NPs was used as a catalyst for the synthesis of propargylamines by the stabilization of copper nanoparticles on the ZSM-5 support. Copper nanoparticles, through their coordination effect, have instantaneous nucleation and slow growth. The ZSM-5/APTMS/(*E*)-4-((pyridin-2-ylimino)methyl)benzaldehyde@Cu-NPs catalyst was applied to perform the response in order to perform aromatic and aliphatic aldehydes and various amines (Fig. 6).

## 6. Comparison of each catalyst sections in the final catalyst

To check whether the modulation of the microenvironment around the Cu NPs affected the promotion of the A^3^-coupling reaction, we monitored the formation of all the synthesized propargylamine derivatives in the presence of Cu NPs decorated ZSM-5, ZSM-5@ATPMS, and ZSM-5/APTMS/(4-pyridine-2-(ylimino)methyl)benzaldehyde)/Cu-NPs. These tests were performed at the optimum conditions (25 mg catalyst, 60 °C, H_2_O, 120 min) to compare the results. Table S1† indicates that each step successfully modified the electronic structure of the chemical surrounding the Cu NPs, resulting in the improvement of the yield of the reaction. Based on this study, ZSM-5@ATPMS and ZSM-5/APTMS/(4-pyridine-2-(ylimino)methyl)benzaldehyde)/Cu-NPs is the optimum catalyst for promoting the preparation of propargylamines *via* the A^3^-coupling reaction, the data are shown in Table S1.† According to the results obtained, the participation of ligands and organic linkers, *i.e.* (4-pyridin-2-(ylimino)methyl)benzaldehyde), and APTMS (3-amino-propyltriethoxysilane), as well as stabilization of Cu nanoparticles in the synthesis and design of the structure of the final mesoporous zeolite catalyst can successfully emerge in the preparation of a high yield product.

## 7. Comparison of the catalyst with other catalysts in the desired reaction

Table 3 compares the catalytic performance of ZSM-5/APTMS/(4-pyridine-2-(ylimino)methyl)benzaldehyde)/Cu-NPs with some of the reported catalysts from the literature for the three-component preparation of propargylamines. This table indicates that our proposed catalyst exhibits one of the highest reported yields. This could be due to the careful modification of the electronic structure of the ZM-5 *via* the modification process with nitrogen-rich ligands. The presence of APTMS and (*E*)-4-((pyridin-2-ylimino)methyl)benzaldehyde alters the electronic structure of ZSM-5, leading to the boosting of the catalytic ability of the Cu NPs in the final composite.

**Table 3:** Comparison of the synthesized catalyst with other catalysts in the three-component A^3^ coupling reaction

Entrance	Catalyst	Amount of catalyst (% mol)	Solvent	*T* (°C)	Time (h)	Yield (%)	Ref.
1	Cu/Al/oxide mesoporous	0.12	Toluene	90	22	94	71
2	Cu@N-rGo	—	—	70	8	76	72
3	Cu@PMO-IL	0.15 mol	CHCl_3_	60	24	97	73
4	CuO/ZnO/Al_2_O_3_ nanocatalyst	0.05 g	—	80	1.5	94	74
5	CuI/HTNT-5	0.02 g	—	80	1.5	96	75
6	CuI supported Amberlyst A-21 in N_2_ atmosphere	10 mol%	Neat	98	8	85	76
7	Eggshell-Cu(ii)-salophen complex	0.2 g	—	80	4	95	77
8	CuNPs/TiO_2_	0.5 mol	—	70	7	90	78
9	Cu(OH)_*x*_–Fe_3_O_4_	0.1 mol	—	120	3	>99	79
10	Cu^I^–USY zeolite catalyst	0.020 g	—	80	15	80	80
11	Silica-CHDA-Cu	0.020 g	—	80	12	92	38
12	PS-PEG-BPy-CuBr_2_ in N_2_ atmosphere	0.05 mol% Cu	—	110	1	88	81
13	CuO NPs	8	Toluene	90	5	87	82
14	Cu-MCM-41	40 mg	—	90–100	1.5	93	83
15	[AQ_2_Cu(ii)]	5	—	100	1	90	84
16	Fe_3_O_4_@TiO_2_/Cu_2_O	(0.01 g)	—	100	1	95	85
17	ZSM-5@APTMS@*E*(-4-((pyridin-2-ylimino)methyl)benzaldehyde@Cu-NPs	25 mg	K_2_CO_3_·H_2_O	60	2	98	This work

## 8. Reusability

**Fig. 7:**
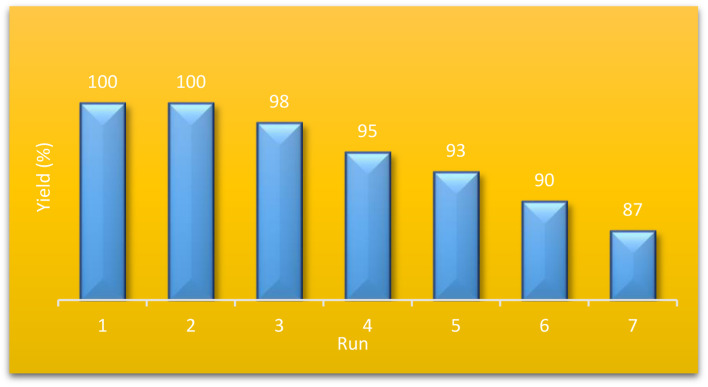
The recyclability of ZSM-5@APTS@(2-aminopyridine/terephthalaldehyde)@Cu-NPs catalyst.

**Fig. 8:**
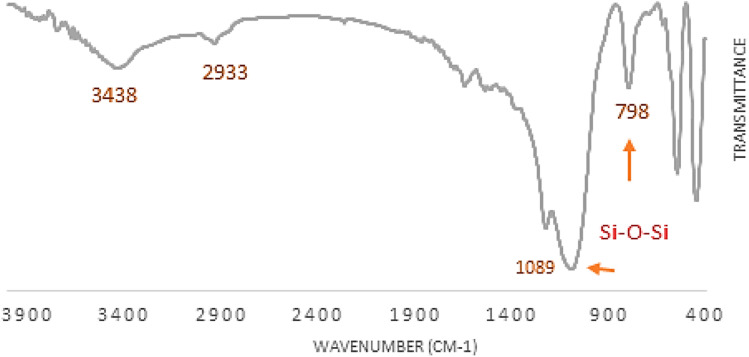
The FT-IR spectrum of the recycled catalyst.

The ability of the catalyst to be used several times in the same reaction is a defining factor for its industrial applications. To investigate the recyclability of our proposed catalyst, we separated the catalyst from the reaction medium by filtration and washed it with ethyl acetate several times for purification. Then, we applied the recycled catalyst in the model reaction for seven cycles and evaluated its performance. As shown in Fig. 7, no decrease in the catalyst performance was observed for four cycles, and even after seven runs, its catalytic performance was still above 90 percent of its first use. This excellent reusability could be due to the high resistance nature of the ZSM-5 in the aqueous medium. Moreover, the modification of the ZSM-5 filled the pores of the MOF, limiting water molecules from accessing the zirconium clusters and increasing their stability. The FT-IR spectrum of recycled catalyst assigned that the catalyst has fully maintained the properties of the catalyst after seven times of use Fig. 8.

### 8.1. ICP-OES analysis

According to the ICP-OES analysis results, the loading of Cu(0) for the fresh catalyst is 0.9. It is mentioned that the ICP analysis of the activities of the recycled catalyst in order to estimate the reusability indicated a very small reduction of less than 0.03% in the amount of copper (Cu) leaching from the synthesized catalyst after seven cycles of recycling. The Cu loading of the catalyst was measured using ICP analysis (Table 4).

**Table 4:** Leaching of Cu

Entrance	Catalyst	(mol%) Cu
1	Order 1	0.9
2	Order 2	0.88
3	Order 3	0.85
4	Order 4	0.8
5	Order 5	0.78
6	Order 6	0.76
7	Order 7	0.75

## 9. Conclusion

In this study, ZSM-5 was chosen as a support for the heterogenization of the Cu NPs due to its high potential for surface area, and inherent structural resistance. The modification of ZSM-5 was carried out by a step-by-step strategy, in which a series of N-rich organic compounds were coordinated to the NH_2_ groups of the organic ligand of the ZSM-5. The resulting catalyst was used to promote the A^3^-coupling reaction, which showed superior performance. The results of this study indicate that such high efficiency is a result of the modulation of the microenvironment of the copper NPs. The proposed catalyst exhibited superior recycling performance due to the inherent resistance of the ZSM-5 and the induced resistance due to its high surface area, interesting channel structure, thermal steadiness, corrosiveness, shape selectivity, and porous properties of ZSM-5.

## Author contributions

L. M. synthesized the final catalyst ZSM-5@(4-pyridine-2-(ylimino)methyl)benzaldehyde)/Cu-NPs catalyst, more synthesized, identified the propargylamine desired. L. M. prepared the manuscript. M. R. V (supervisor or project), M. H (supervisor or project), and S. R. (advisor) reviewed the manuscript.

## Conflicts of interest

The authors declare no competing interests.

## Supplementary Material

RA-013-D2RA07700K-s001
