# Supplementary material for: Stabilization of copper nanoparticles onto the double Schiff-base-functionalized ZSM-5 for A3 coupling reaction catalysis aimed under mild conditions
Source: RSC Adv. 2023 Feb 7;13(7):4843–58. doi: 10.1039/d2ra07700k (PMC9903180; doi:10.1039/d2ra07700k)
Supplement: RA-013-D2RA07700K-s001 [file RA-013-D2RA07700K-s001.pdf]

Electronic Supplementary Material (ESI) for RSC Advances.
This journal is © The Royal Society of Chemistry 2023

Electronic Supporting information

Stabilization of copper nanoparticles onto the double Schiff-base-functionalized ZSM-5 for A^3 coupling reaction catalysis aiming under mild conditions

Leila Mohammadi[a], Mojtaba Hosseinifard[b*], Mohammad Reza Vaezi[a*], Sadegh Rostamnia[c]

[a]*Department of Nano Technology and Advanced Materials, Materials and Energy Research Center, Karaj, Iran. E-mail: l.mohammadi3790@gmail.com,* m_r_vaezi@merc.ac.ir

[b]*Department of Energy, Materials and Energy Research Center, Karaj, Iran. E-mail: m.hosseini@merc.ac.ir*

[c] *Organic and Nano Group (ONG), Department of Chemistry, Iran University of Science and Technology (IUST), PO BOX 16846-13114, Tehran, Iran. E-mail: rostamnia@iust.ac.ir*

Table S1. investigation of the effects on catalyst efficiency

Entrance	Product	Time (min)	ZSM-5@Cu Yield (%)	ZSM-5@ATPS@Cu Yield (%)	ZSM-5@ATPS@2-aminopyridine/ terephthalaldehyde@Cu Final Catalyst Yield (%)
**1**	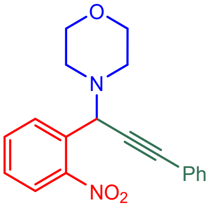	120	85	88	**99**
**2**	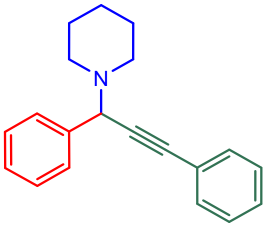	120	82	85	**98**
**3**	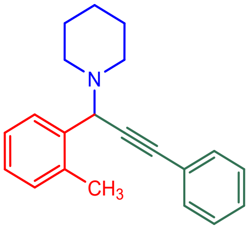	120	76	79	**91**
**4**	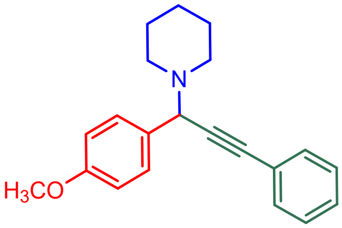	120	75	77	**90**
**5**	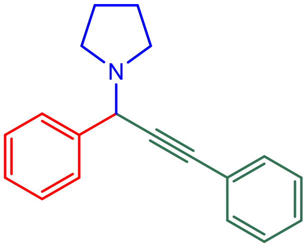	120	77	80	**93**
**6**	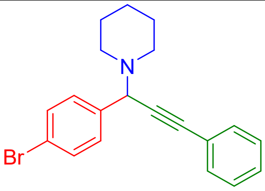	120	81	83	**95**

**7**	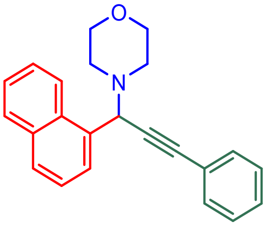	120	80	81	**92**
**8**	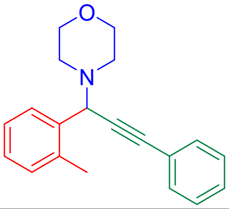	120	78	79	**91**
**9**	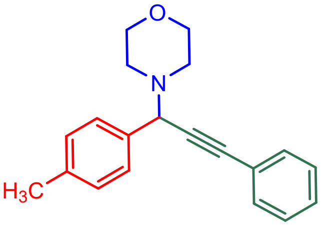	120	78	79	**92**
**10**	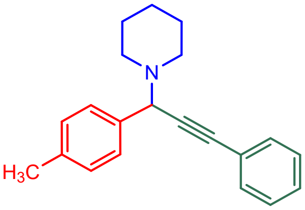	120	80	81	**94**
**11**	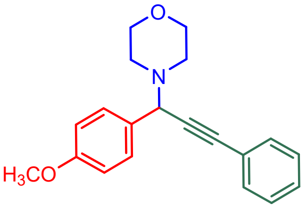	120	78	79	**90**
**12**	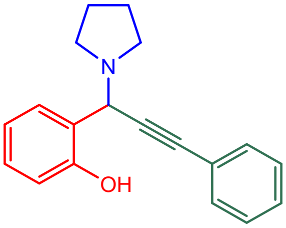	120	84	85	**95**
**13**	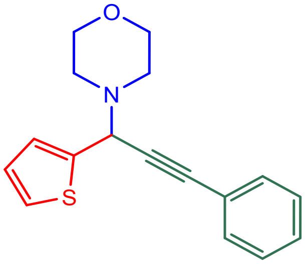	120	75	76	**93**

**14**	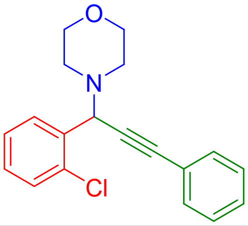	120	76	77	**92**
**15**	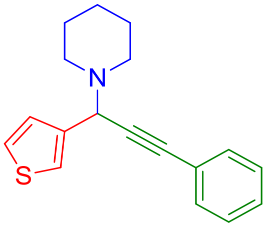	120	79	79	**94**
**16**	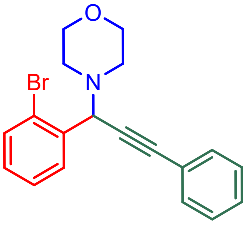	120	79	79	**93**
**17**	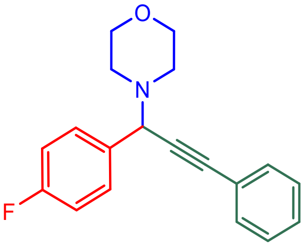	120	83	84	**95**
**18**	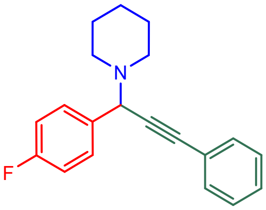	120	84	85	**96**
**19**	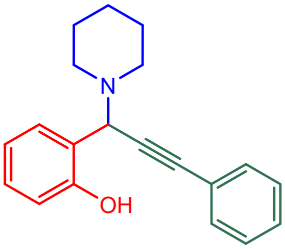	120	85	85	**97**
**20**	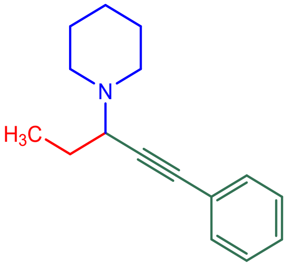	120	67	68	**85**

Reaction conditions: 1 mmol of morpholine, 1 mmol of benzaldehyde, and 1.1 mmol of phenylacetylene.

1-(1-(2-nitrophenyl)-3-phenylprop-2-ynyl) morpholine

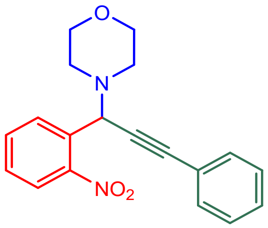

1 H NMR (400 MHz, $CDCl_3$): δ 2.43 (s, 2H), 2.62 (s, 2H), 3.63 (s, 4H), 5.65 (s, 1H), 7.26-7.56 (m, 7H), 7.71 (d, J = 7.5 Hz, 1H), 7.95 (d, J = 7.2 Hz, 1H). 13 C NMR (100 MHz, $CDCl_3$): δ 49, 57.3, 66.3, 81.4, 90.1, 121.9, 123.8, 127.9, 128.1, 128.3, 129.6, 130.9, 131.3, 131.6, 149.4.

2-(1,3-diphenylprop-2-yn-1-yl) piperidine

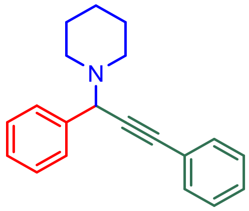

^{1}H NMR ($CDCl_3$, 400 MHz): δ 7.70-7.64 (m, 2H), 7.58-7.53 (m, 2H), 7.43-7.34 (m, 6H), 4.91 (s, 1H), 2.65 (t, 4H), 1.73-1.59 (m, 4H), 1.50-1.48 (m, 2H). ^{13}C NMR ($CDCl_3$, 100 MHz): δ 138.00, 131.85, 128.74, 128.33, 128.19, 128.15, 127.68, 123.20, 88.08, 85.69, 62.33, 50.63, 25.94, 24.31.

3-(3-phenyl-1-(p-tolyl) prop-2-yn-1-yl) piperidine

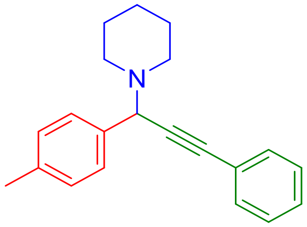

^{1}H NMR ($CDCl_3$, 400 MHz): δ 7.59-7.54 (m, 4H), 7.39-7.35 (m, 3H), 7.22 (d, 2H, J=8), 4.89 (s, 1H), 2.66 (t, 4H), 2.40 (s, 3H), 1.73-1.60 (m, 4H), 1.51-1.49 (m, 2H). ^{13}C NMR ($CDCl_3$, 100 MHz): δ 137.40, 134.90, 131.85, 128.87, 128.72, 128.33, 128.16, 123.27, 87.90, 85.96, 62.08, 50.61, 25.92, 24.34, 21.19.

4-(1-(4-methoxyphenyl)-3-phenylprop-2-yn-1-yl) piperidine

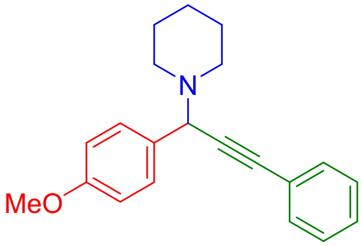

^{1}H NMR ($CDCl_3$, 400 MHz): δ 7.59-7.55 (m, 4H), 7.38-7.36 (m, 3H), 6.94 (d, 2H, J=8), 4.80 (s, 1H), 3.86 (s, 3H), 2.60 (br, 4H), 1.67-1.47 (m, 6H). ^{13}C NMR ($CDCl_3$, 100 MHz): δ 159.00, 131.83, 130.67, 129.71, 128.31, 128.05, 123.41, 113.41, 87.65, 86.43, 61.80, 55.31, 50.64, 26.20, 24.51.

5-(1,3-diphenylprop-2-yn-1-yl) pyrrolidine

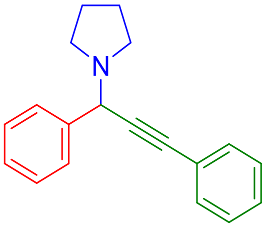

^{1}H NMR ($CDCl_3$, 400 MHz): δ 7.66 (t, 2H), 7.55-7.53 (m, 2H), 7.45-7.34 (m, 6H), 5.06 (s, 1H), 2.85-2.72 (m, 4H), 2.03-1.82 (m, 4H). ^{13}C NMR ($CDCl_3$, 100 MHz): δ 138.69, 131.84, 128.51, 128.37, 128.34, 128.03, 127.87, 123.03, 87.29, 86.07, 58.97, 50.19, 23.52.

6-(1-(4-bromophenyl)-3-phenylprop-2-yn-1-yl) piperidine

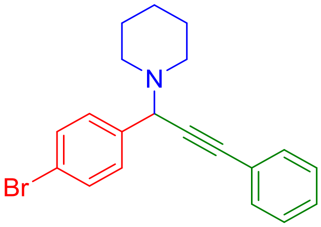

^{1}H NMR ($CDCl_3$, 400 MHz): δ 7.69 (d, 1H, J=8), 7.58-7.50 (m ,5H), 7.37 (t, 3H), 4.79 (s, 1H), 2.58 (t, 4H), 1.70-1.57 (m, 4H), 1.50-1.38 (m, 2H). ^{13}C NMR ($CDCl_3$, 100 MHz): δ 137.83, 131.85, 131.18, 130.24, 129.06, 128.36, 128.26, 88.30, 85.29, 61.79, 50.67, 26.14, 24.39.

7-(1-(naphthalen-3-yl)-3-phenylprop-2-ynyl) piperidine

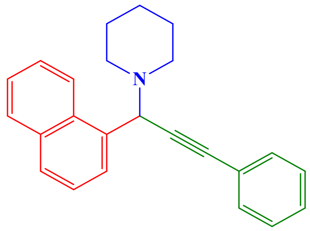

^{1}H NMR (400 MHz, $CDCl_3$, ppm): δ 1.47-1.51 (m, 2H), 1.60-1.67 (m, 4H), 2.64 (t, 4H), 4.97 (s, 1H), 7.36-7.40 (m, 3H), 7.48-7.52 (m, 2H), 7.58-7.61 (m, 2H), 7.79 (dd, $J^1=J^2$=8.4 Hz, 1H), 7.85-7.91 (m, 3H), 8.11 (s, 1H); ^{13}C NMR (100 MHz, $CDCl_3$, ppm) d 24.4, 26.2, 50.8, 62.5, 86, 88.1, 123.3, 125.8, 125.9, 126.7, 127.2, 127.5, 127.7, 128.1, 128.12, 131.8, 132.9, 133.1, 136.3.

8-(3-phenyl-1-(o-tolyl) prop-2-yn-1-yl) morpholine

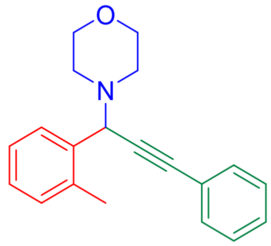

^{1}H NMR (400 MHz, Chloroform-*d*) δ 7.76 – 7.71 (m, 1H), 7.58 – 7.53 (m, 2H), 7.39 – 7.32 (m, 3H), 7.23 (dd, *J* = 5.8, 3.7 Hz, 3H), 4.91 (s, 1H), 3.77 – 3.64 (m, 4H), 2.66 (t, *J* = 4.7 Hz, 4H), 2.51 (s, 3H). ^{13}C NMR (75 MHz, Chloroform-d) δ 137.6, 135.8, 131.8, 130.8, 129.1, 128.4, 128.2, 127.9, 125.4, 123.1, 88.7, 85.1, 67.3, 59.9, 49.8, 19.2.

9-(3-phenyl-1-(p-tolyl) prop-2-yn-1-yl) morpholine

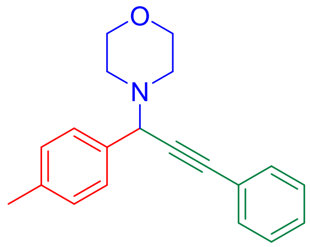

^{1}H NMR (400 MHz, Chloroform-*d*) δ 7.50 (q, *J* = 3.5 Hz, 4H), 7.31 (t, *J* = 4.6 Hz, 3H), 7.20 – 7.13 (m, 2H), 4.74 (d, *J* = 3.4 Hz, 1H), 3.72 (d, *J* = 4.9 Hz, 4H), 2.62 (t, *J* = 4.6 Hz, 4H), 2.34 (s,3H).^{13}C NMR (100 MHz, Chloroform-d) δ 137.5, 134.8, 131.8, 129.0, 128.6, 128.3, 128.2, 123.0, 88.3, 85.34, 67.2, 61.8, 49.9, 21.2.

10-(3-phenyl-1-(p-tolyl) prop-2-yn-1-yl)piperidine

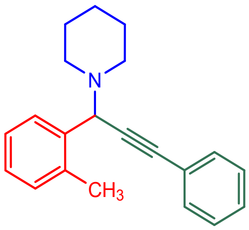

^1H NMR ($CDCl_3$, 400 MHz): δ 7.59-7.54 (m, 4H), 7.39-7.35 (m, 3H), 7.22 (d, 2H, J=8), 4.89 (s, 1H), 2.66 (t, 4H), 2.40 (s, 3H), 1.73-1.60 (m, 4H), 1.51-1.49 (m, 2H). ^{13}C NMR ($CDCl_3$, 100 MHz): δ 137.40, 134.90, 131.85, 128.87, 128.72, 128.33, 128.16, 123.27, 87.90, 85.96, 62.08, 50.61, 25.92, 24.34, 21.19.

11-(1-(4-methoxyphenyl)-3-phenylprop-2-yn-1-yl) morpholine

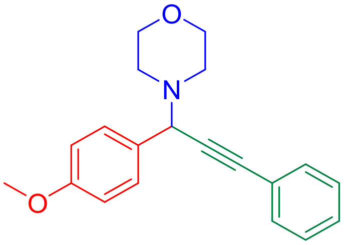

^1H NMR (300 MHz, Chloroform-*d*) δ 7.57 (t, J = 1.2 Hz, 1H), 7.54 (ddd, J = 6.0, 2.5, 1.6 Hz, 3H), 7.37 – 7.31 (m, 3H), 6.95 – 6.89 (m, 2H), 4.76 (s, 1H), 3.81 (s, 3H), 3.75 (td, J = 5.1, 3.0 Hz, 4H), 2.64 (dt, J = 5.2, 3.3 Hz, 4H). ^{13}C NMR (75 MHz, Chloroform-*d*) δ 159.2, 131.8, 129.8, 129.7, 128.3, 128.2, 123.0, 113.6, 88.3, 85.40, 67.1, 61.4, 55.3, 49.8.

12-(3-phenyl-1-(pyrrolidin-1-yl) prop-2-ynyl) phenol

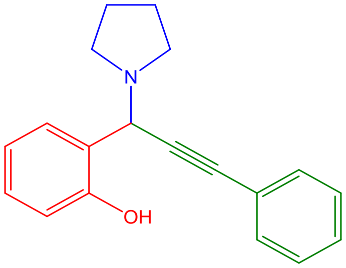

^1H NMR (400 MHz, $CDCl_3$): δ 1.87-1.89 (m, 4H), 2.78-2.88 (m, 4H), 5.29 (s, 1H), 6.82-6.87 (m, 2H), 7.19-7.27 (m, 1H), 7.34-7.37 (m, 3H), 7.51-7.54 (m, 3H). 13 C NMR (100 MHz, $CDCl_3$): δ 23.8, 48.9, 57.1, 83, 89, 116.2, 118.9, 122.2, 122.6, 127.8, 128.4, 128.5, 128.8, 129.9, 131.9, 157.6.

13-(3-phenyl-1-(thiophen-2-yl) prop-2-yn-1-yl)morpholine

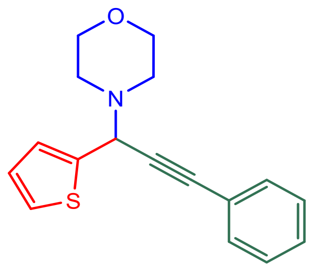

^{1}H NMR ($CDCl_3$, 400 MHz): δ 7.57-7.53 (m, 2H), 7.38-7.28 (m, 5H), 7.02-7.00 (m, 1H), 5.05 (s, 1H), 3.84-3.77 (m, 4H), 2.79-2.68 (m, 4H). ^{13}C NMR ($CDCl_3$, 100 MHz): δ 142.66, 131.89, 128.49, 128.38, 126.51, 126.38, 125.89, 122.66, 87.64, 84.25, 67.12, 57.79, 49.64.

14-(1-(2-chlorophenyl)-3-phenylprop-2-yn-1-yl) morpholine

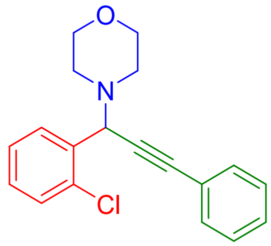

^{1}H NMR ($CDCl_3$, 400 MHz): δ 7.81 (d, 1H, *J*=8), 7.56-7.53 (m, 2H), 7.48-7.42 (m, 1H), 7.39-7.28 (m, 5H), 5.18 (s, 1H), 3.79-3.75 (m, 4H), 2.73 (br, 4H). ^{13}C NMR ($CDCl_3$, 100 MHz): δ 135.51, 134.68, 131.86, 130.62, 129.95, 129.22, 128.44, 128.38, 126.42, 122.78, 88.42, 84.65, 67.11, 58.96, 49.85.

15-(3-phenyl-1-(thiophen-3-yl) prop-2-yn-1-yl) piperidine:

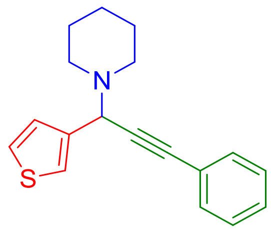

^{1}H NMR ($CDCl_3$, 400 MHz): δ 7.57-7.55 (m, 2H), 7.46 (s, 1H), 7.41-7.29 (m, 5H), 4.88 (s, 1H), 2.62 (br, 4H), 1.83-1.60 (m, 4H), 1.51-1.49 (m, 2H). ^{13}C NMR ($CDCl_3$, 100 MHz): δ 140.29, 131.86, 128.34, 128.15, 127.94, 125.39, 123.29, 123.26, 86.68, 86.22, 58.31, 50.62, 26.20, 24.48.

1-(1-(2-nitrophenyl)-3-phenylprop-2-yn-1-yl) morpholine

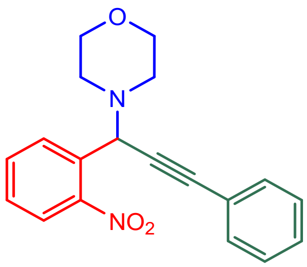

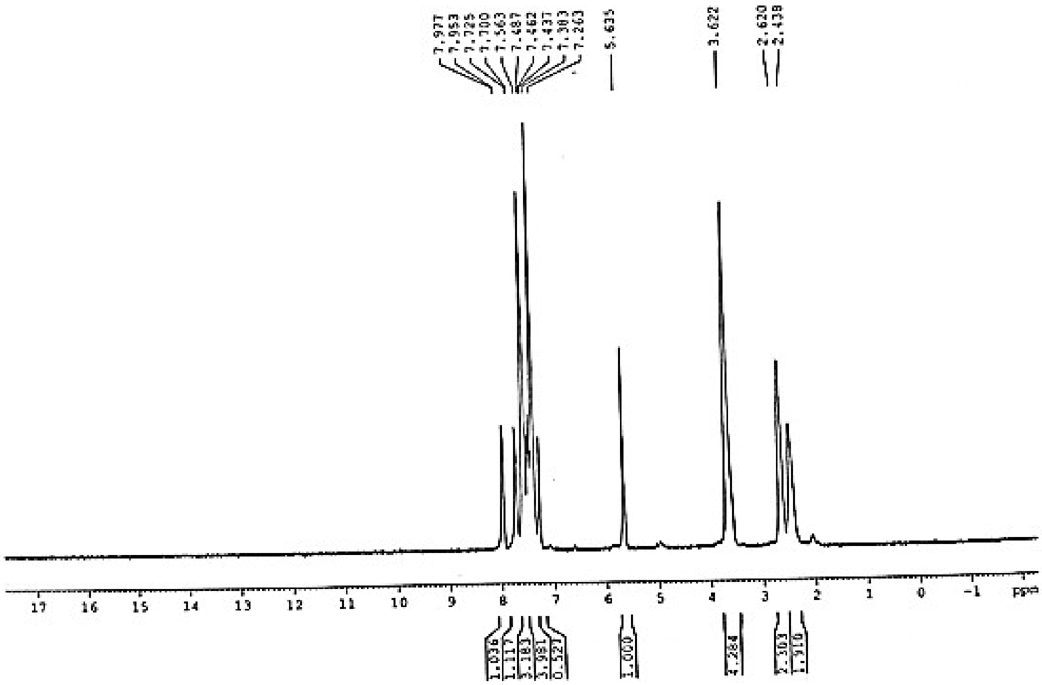

^{1}HNMR

4-(1-(2-nitrophenyl)-3-phenylprop-2-yn-1-yl)morpholine

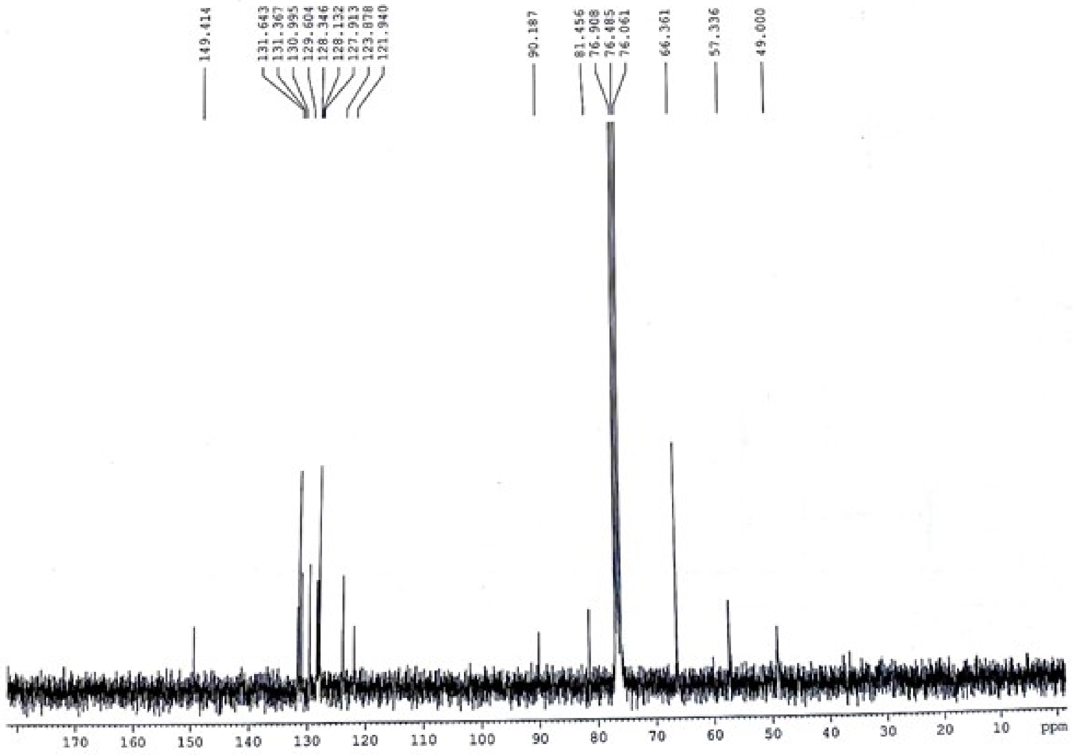

^{13}CNMR

4-(1-(2-nitrophenyl)-3-phenylprop-2-yn-1-yl) morpholine

2 -(1,3-diphenylprop-2-yn-1-yl) piperidine

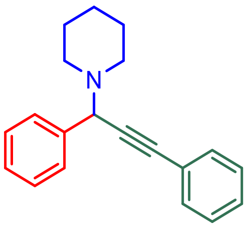

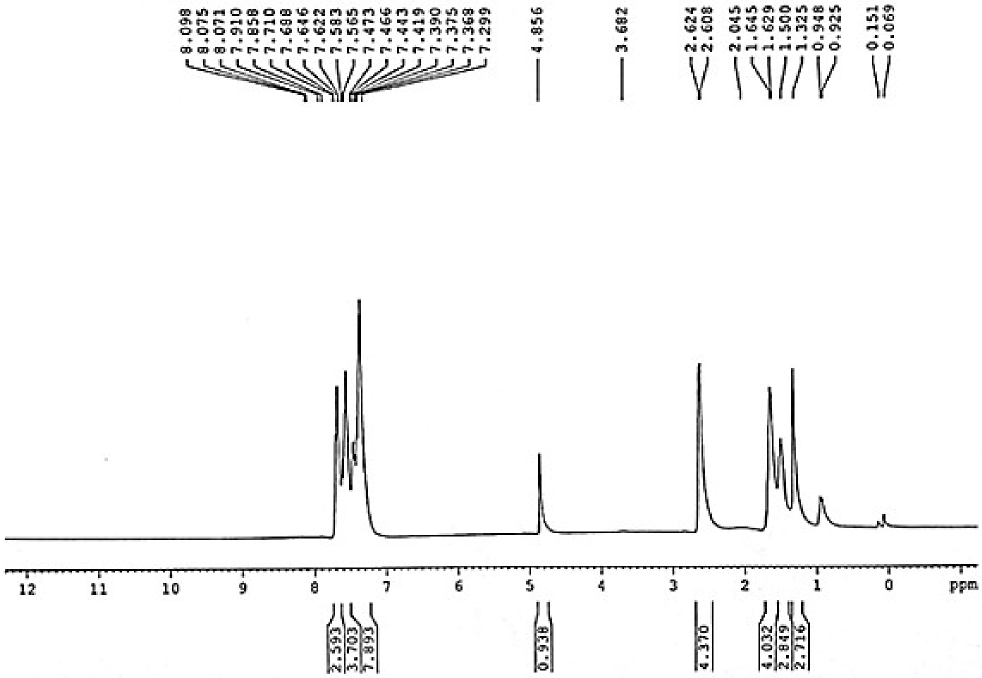

^{1}HNMR

1-(1,3-diphenylprop-2-yn-1-yl) piperidine

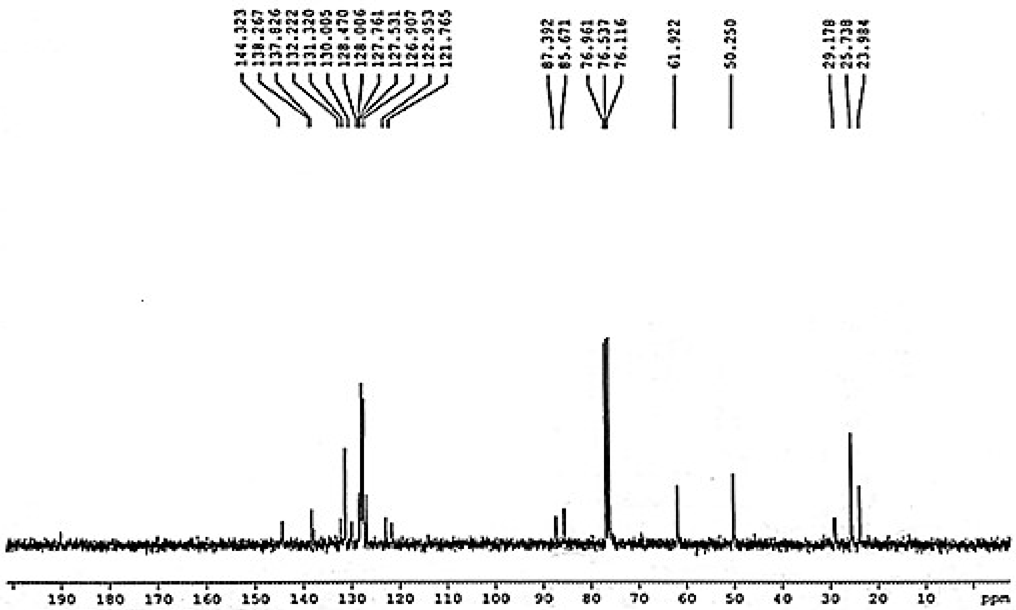

^{13}CNMR

1-(1,3-diphenylprop-2-yn-1-yl) piperidine

3-(3-phenyl-1-(p-tolyl) prop-2-yn-1-yl)piperidine

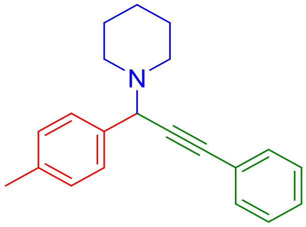

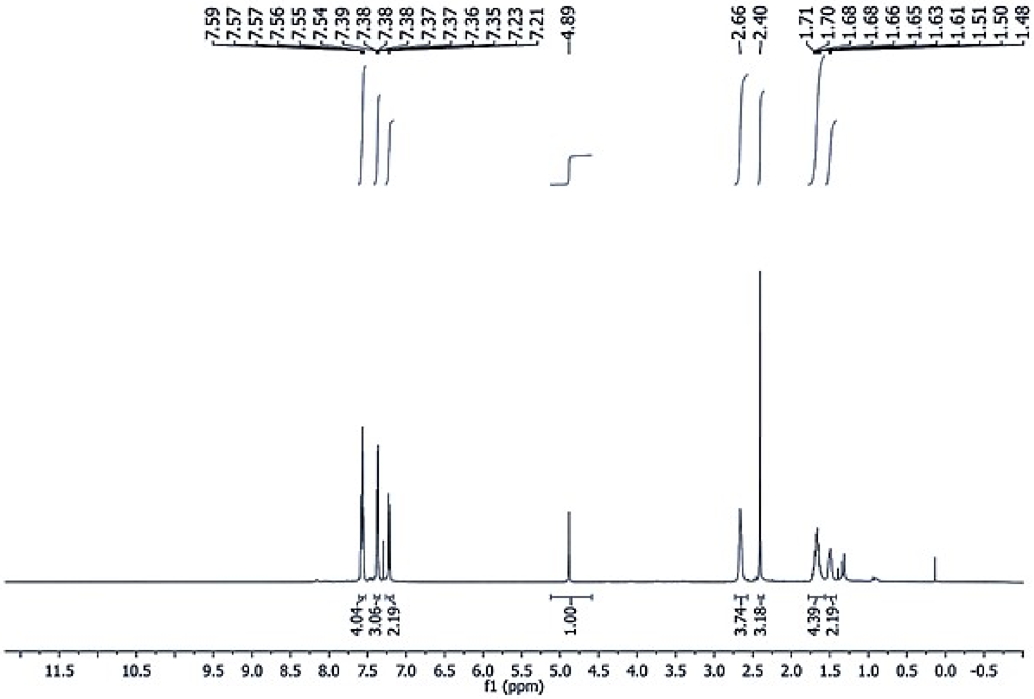

^{1}H NMR

1-(3-phenyl-1-(p-tolyl) prop-2-yn-1-yl)piperidine

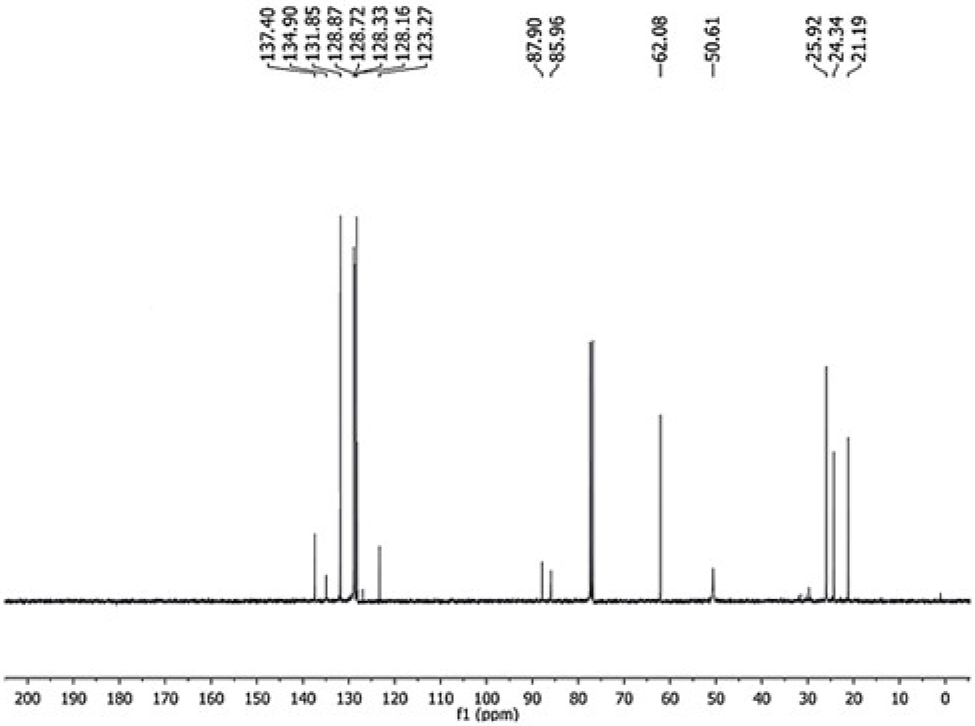

^{13}C NMR

1-(3-phenyl-1-(p-tolyl) prop-2-yn-1-yl)piperidine

4-(1-(4-methoxyphenyl)-3-phenylprop-2-yn-1-yl) piperidine

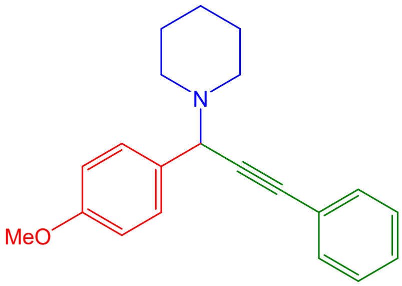

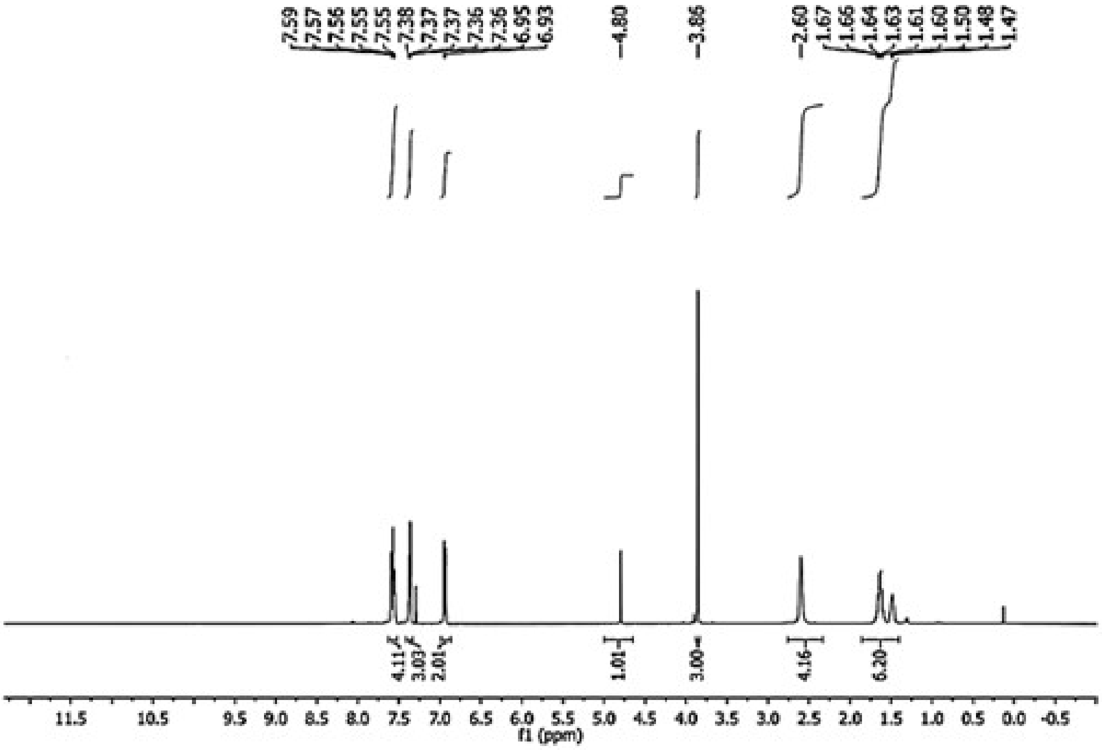

^{1}H NMR

1-(1-(4-methoxyphenyl)-3-phenylprop-2-yn-1-yl) piperidine

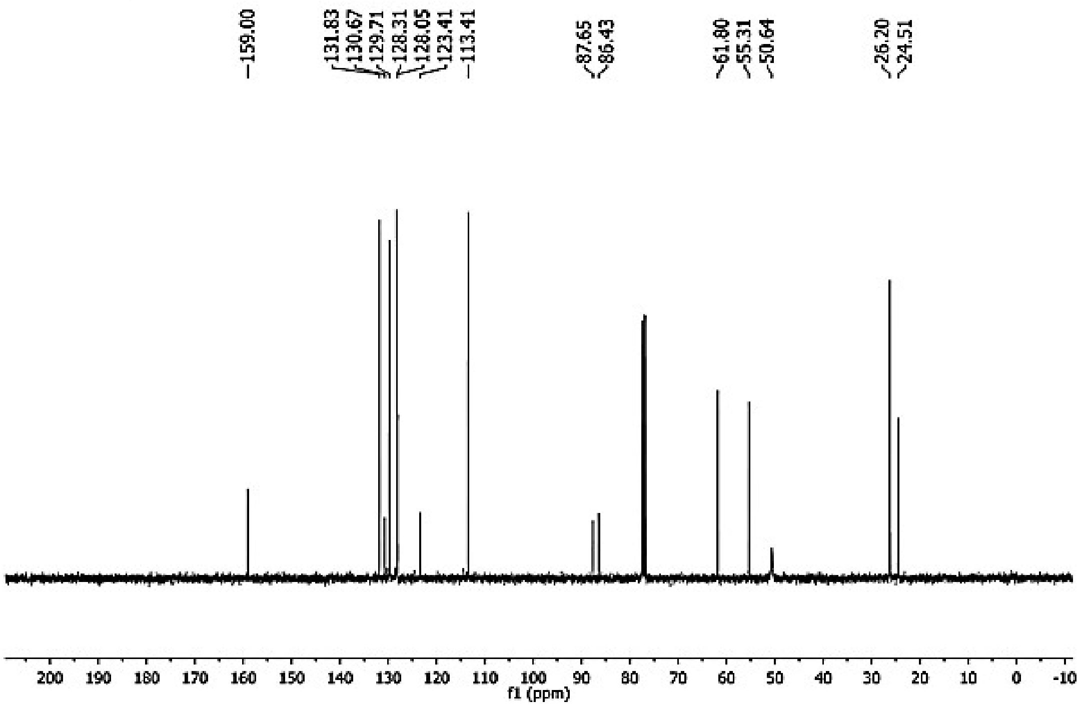

^{13}CNMR

1-(1-(4-methoxyphenyl)-3-phenylprop-2-yn-1-yl) piperidine

5. 1-(1,3-diphenylprop-2-yn-1-yl) pyrrolidine

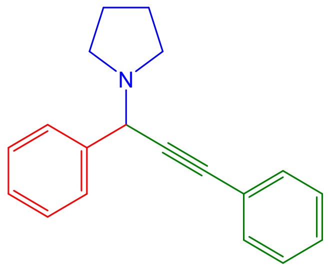

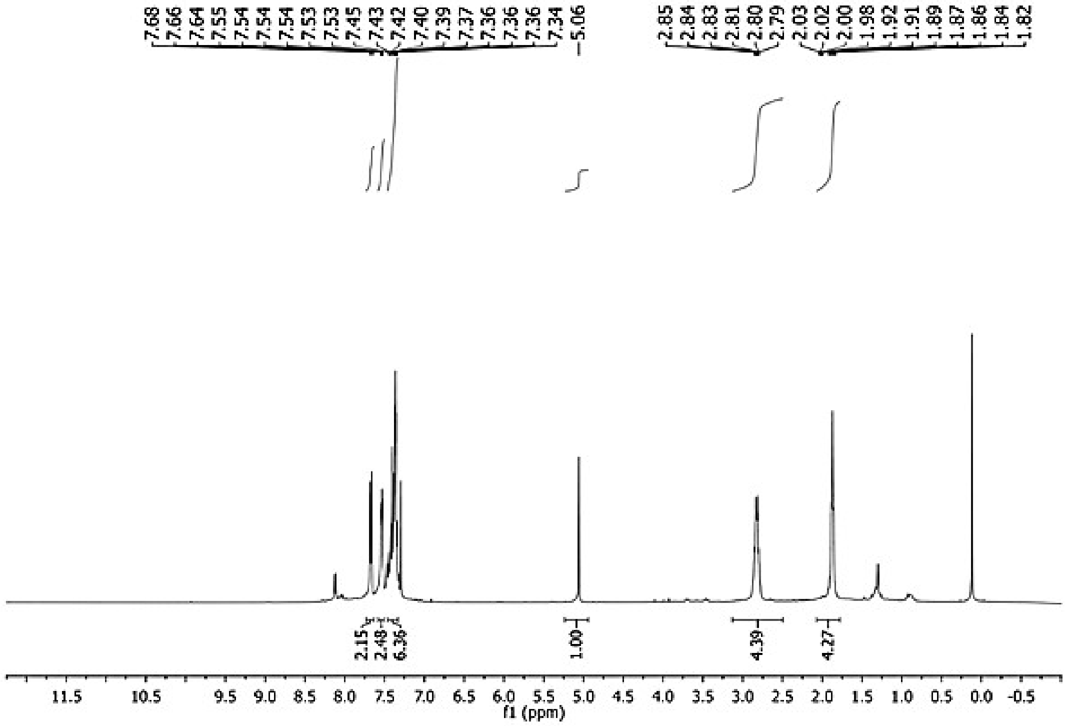

^{1}H NMR

1-(1,3-diphenylprop-2-yn-1-yl) pyrrolidine

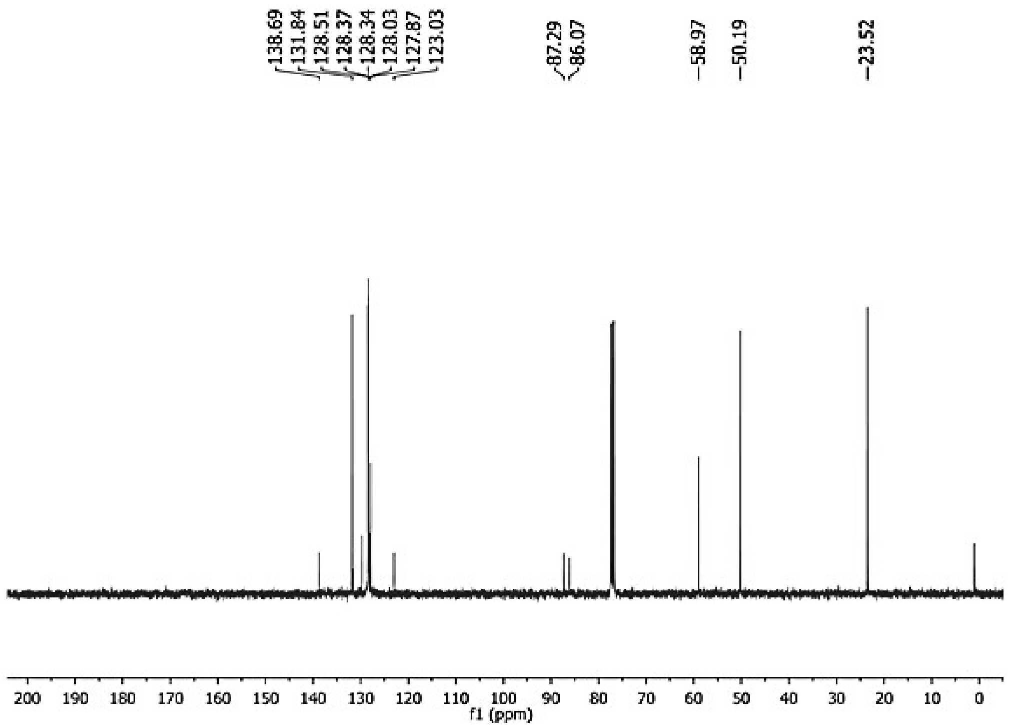

^{13}C NMR

1-(1,3-diphenylprop-2-yn-1-yl) pyrrolidine

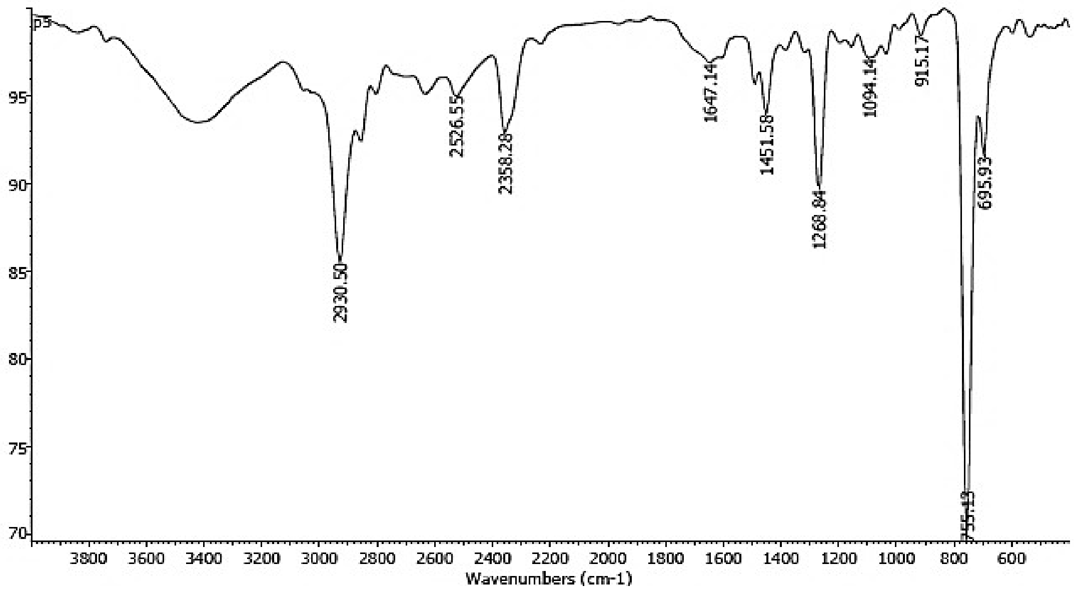

FT-IR

1-(1,3-diphenylprop-2-yn-1-yl) pyrrolidine

6-(1-(4-bromophenyl)-3-phenylprop-2-yn-1-yl) piperidine

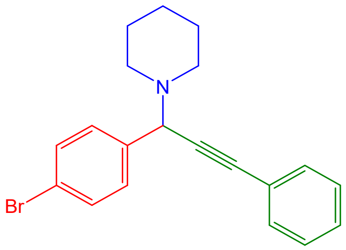

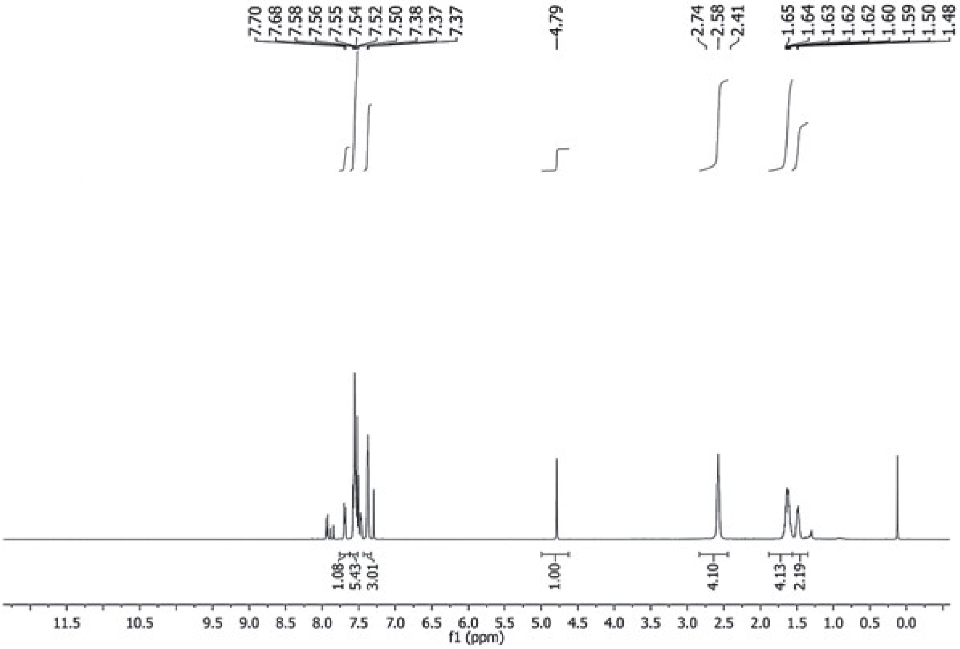

^{1}H NMR

1-(1-(4-bromophenyl)-3-phenylprop-2-yn-1-yl) piperidine

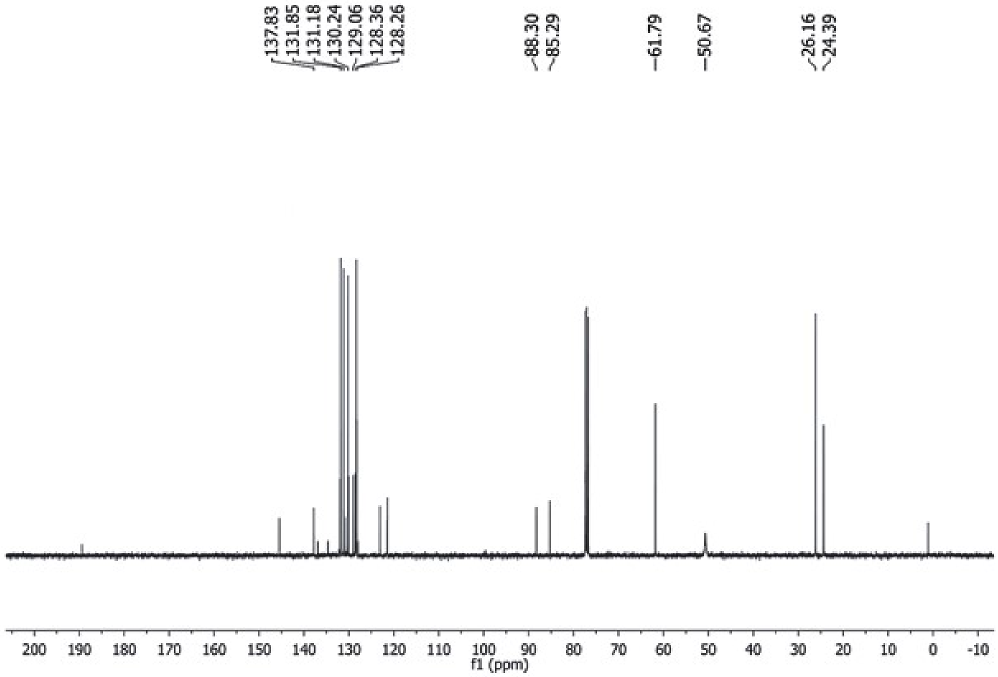

^{13}C NMR

1-(1-(4-bromophenyl)-3-phenylprop-2-yn-1-yl) piperidine

7-(1-(naphthalen-1-yl)-3-phenylprop-2-yn-1-yl) piperidine

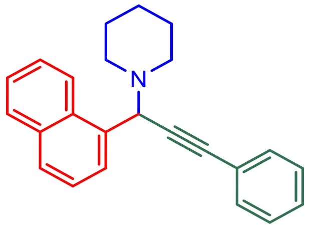

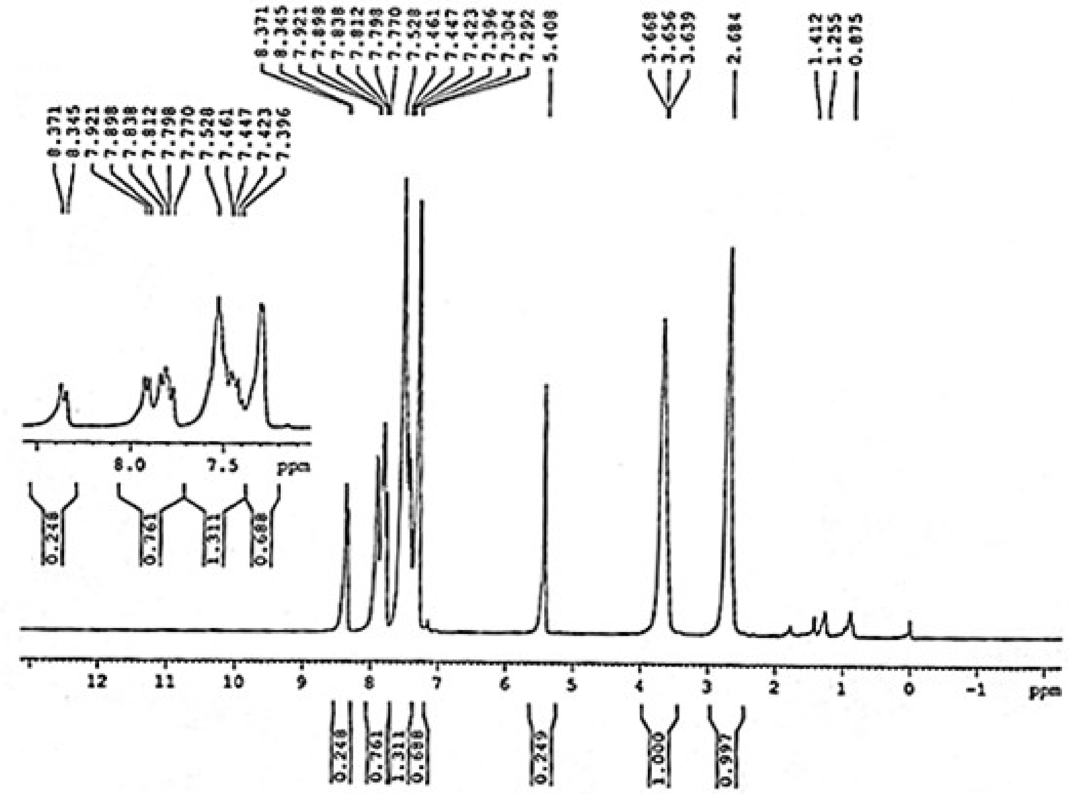

^{1}H NMR

1-(1-(naphthalen-1-yl)-3-phenylprop-2-yn-1-yl) piperidine

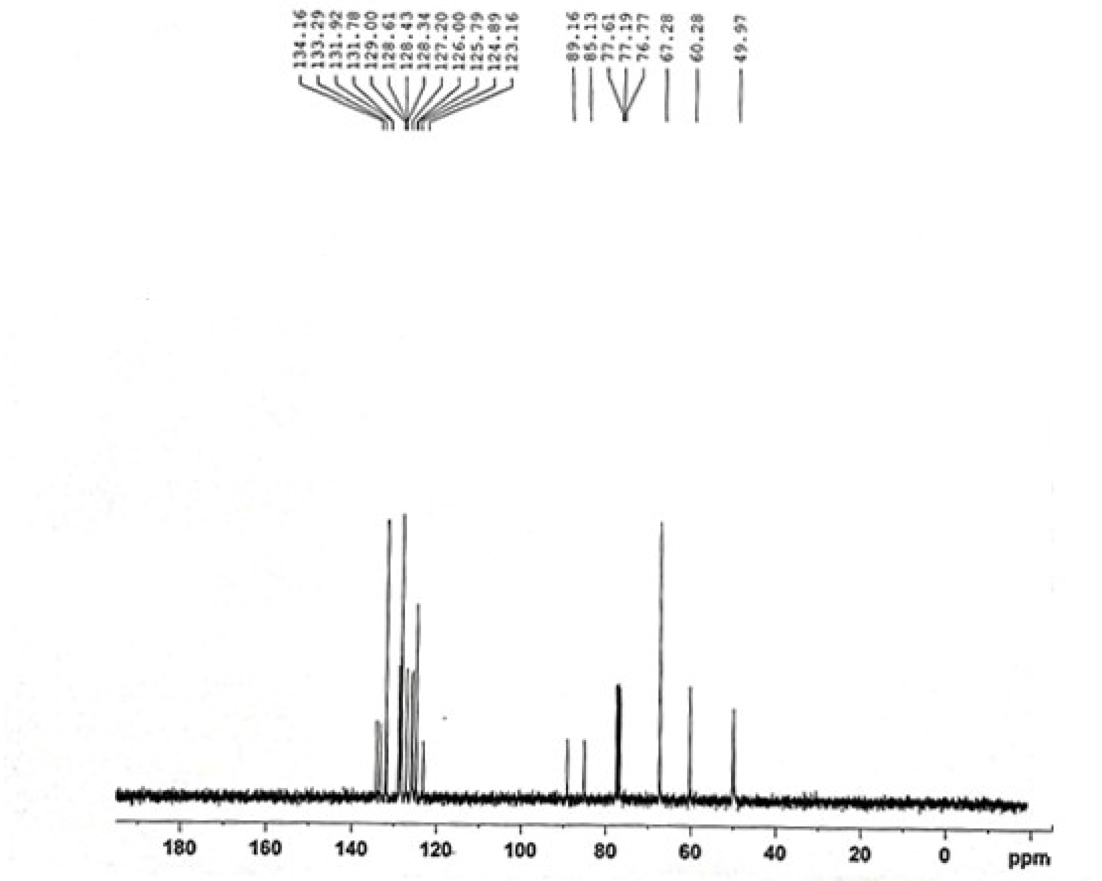

^{13}C NMR

1-(1-(naphthalen-1-yl)-3-phenylprop-2-yn-1-yl) piperidine

8-(3-phenyl-1-(o-tolyl) prop-2-yn-1-yl) morpholine

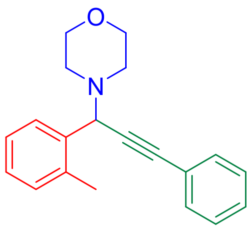

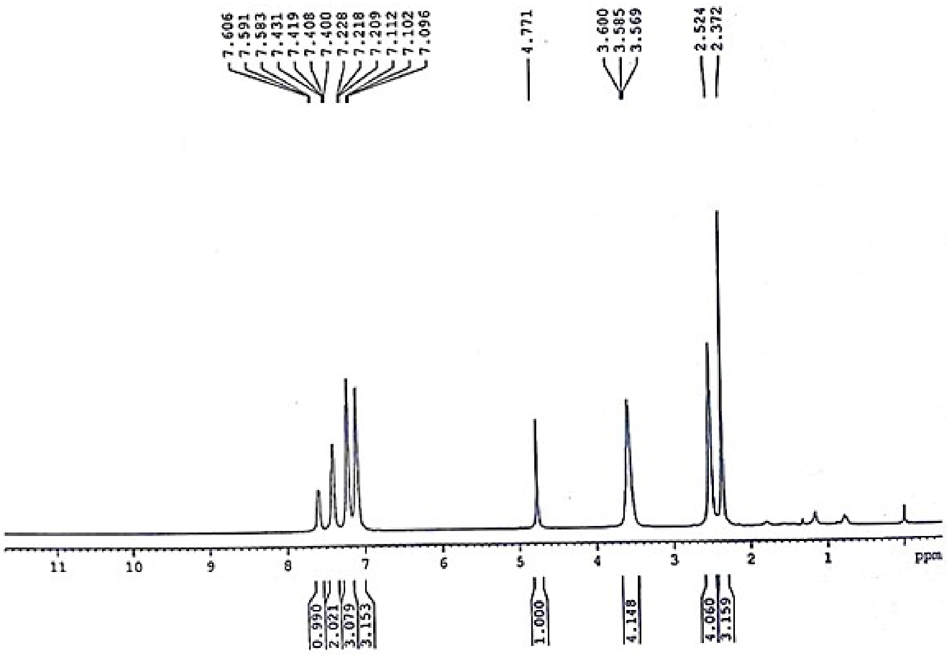

^{1}H NMR

4-(3-phenyl-1-(o-tolyl) prop-2-yn-1-yl) morpholine

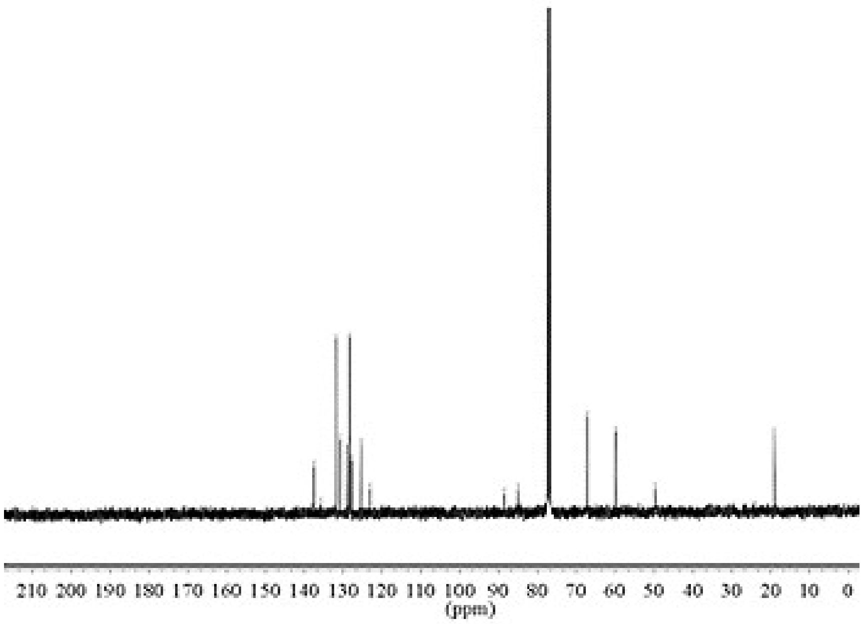

^{13}C NMR

4-(3-phenyl-1-(o-tolyl) prop-2-yn-1-yl) morpholine

9-(3-phenyl-1-(p-tolyl) prop-2-yn-1-yl)morpholine

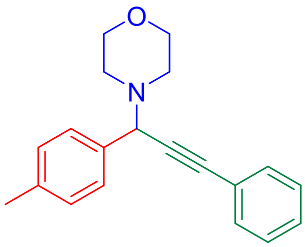

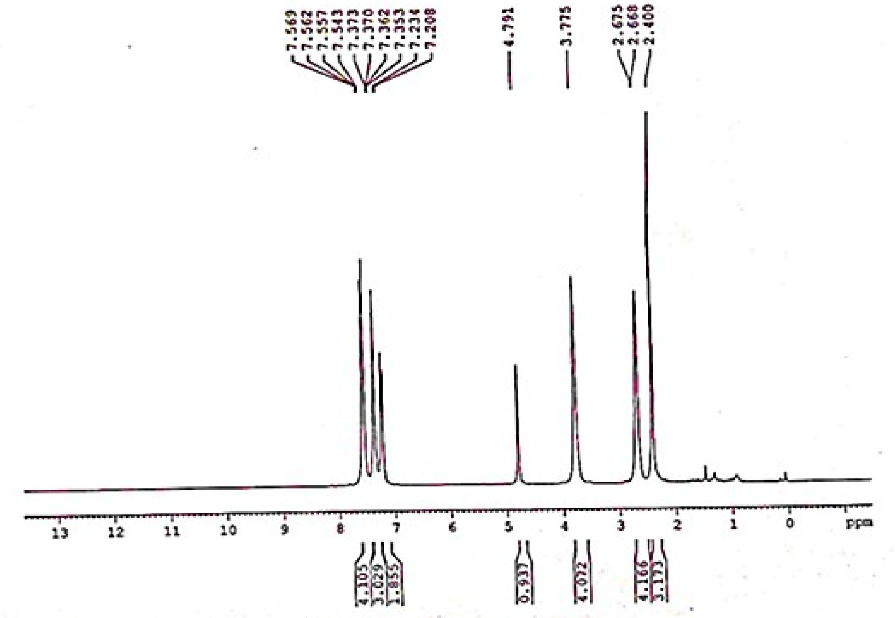

^{1}H NMR

4-(3-phenyl-1-(p-tolyl) prop-2-yn-1-yl)morpholine

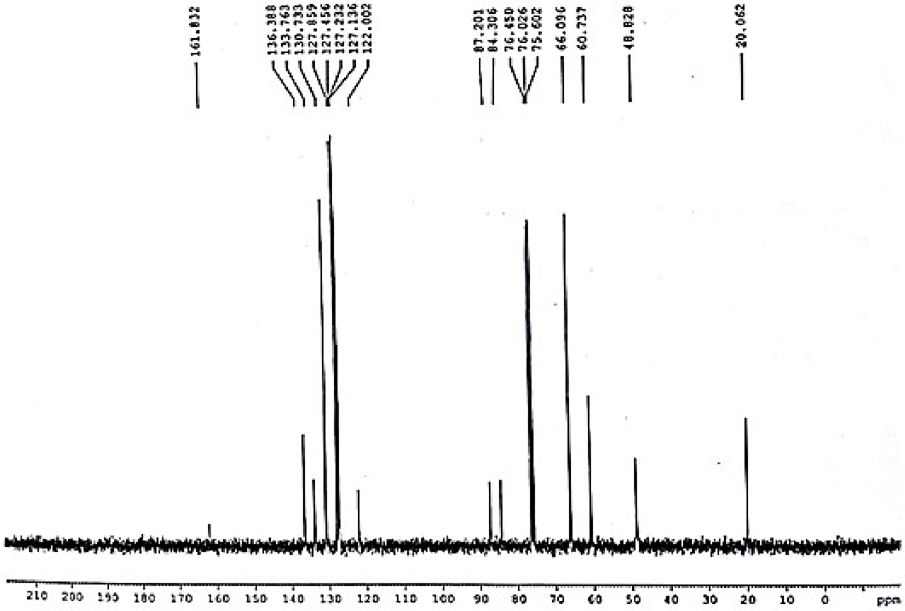

^{13}C NMR

4-(3-phenyl-1-(p-tolyl) prop-2-yn-1-yl) morpholine

10-(3-phenyl-1-(o-tolyl) prop-2-yn-1-yl)piperidine

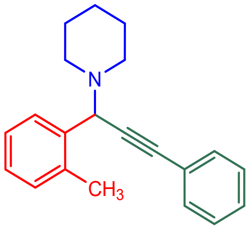

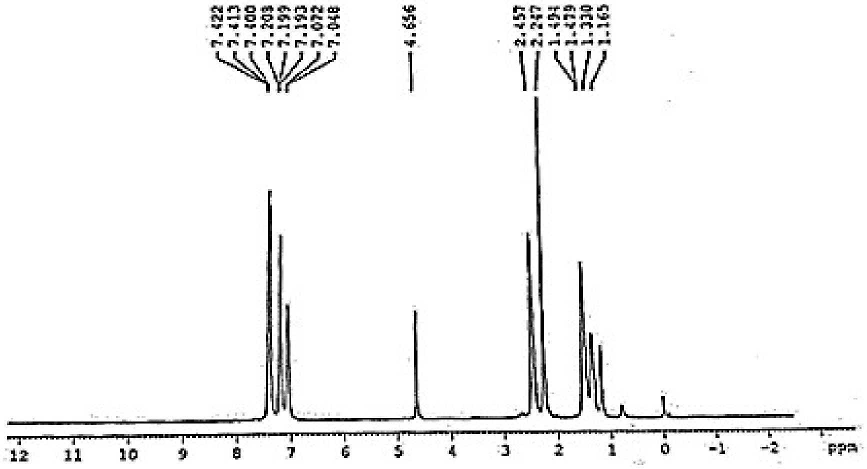

^{1}H NMR

1-(3-phenyl-1-(o-tolyl) prop-2-yn-1-yl)piperidine

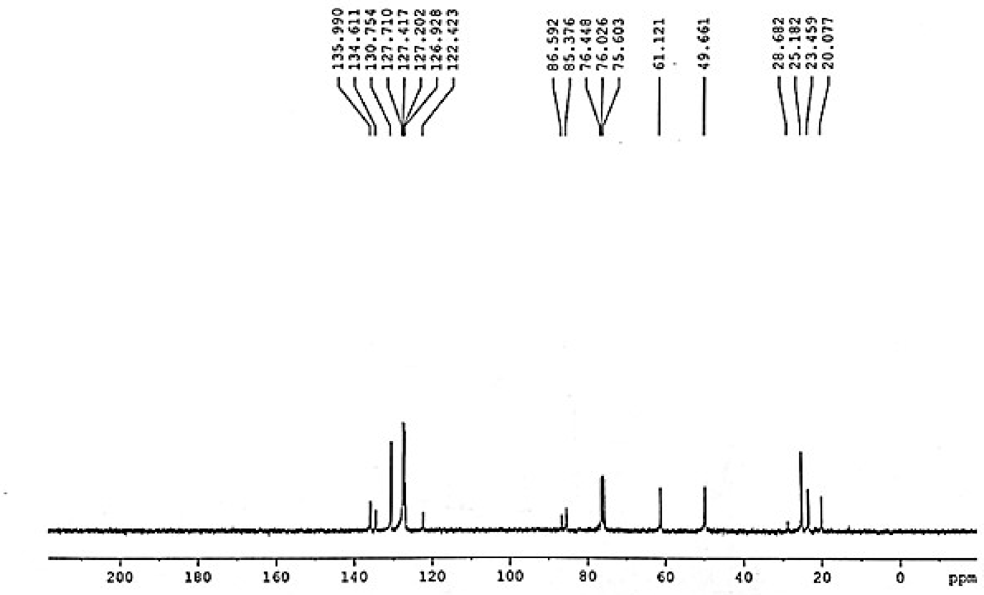

^{13}C NMR

1-(3-phenyl-1-(o-tolyl) prop-2-yn-1-yl)piperidine

11-(1-(4-methoxyphenyl)-3-phenylprop-2-yn-1-yl) morpholine

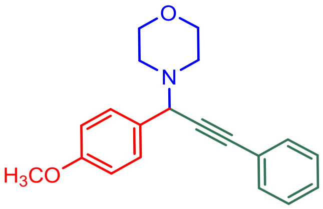

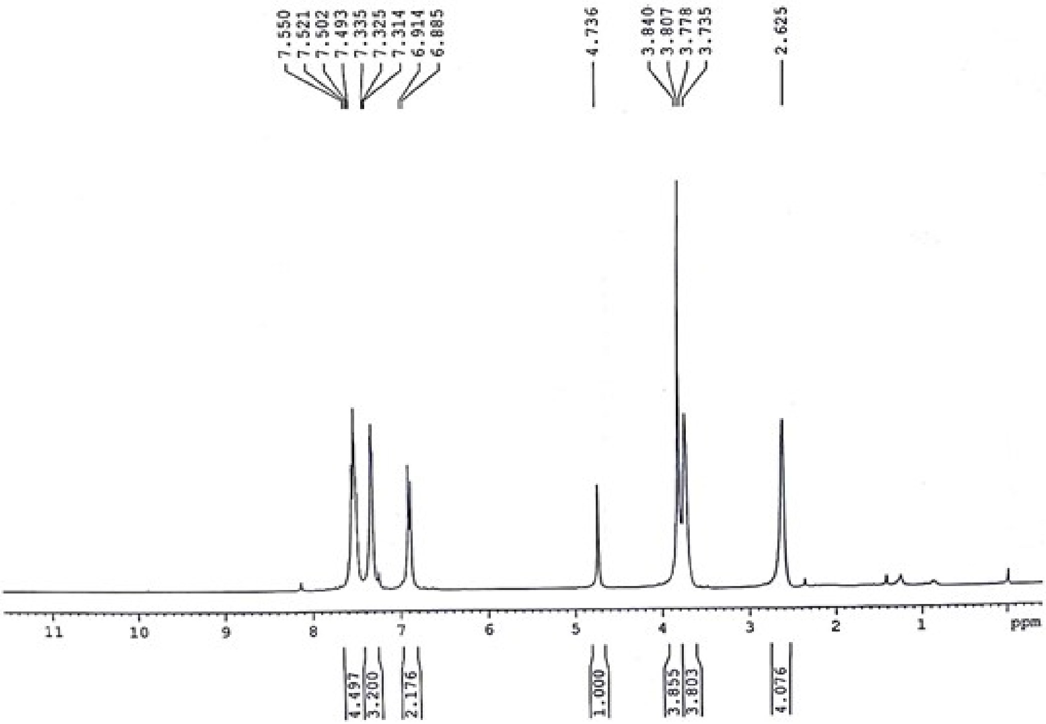

^{1}H NMR

4-(1-(4-methoxyphenyl)-3-phenylprop-2-yn-1-yl) morpholine

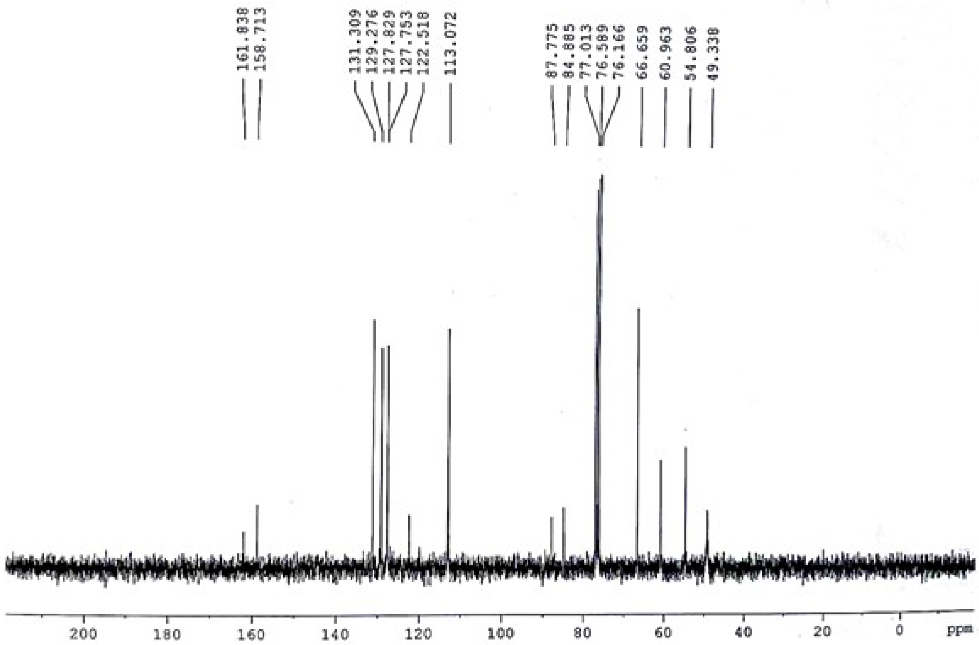

^{13}C NMR

4-(1-(4-methoxyphenyl)-3-phenylprop-2-yn-1-yl) morpholine

12-(3-phenyl-1-(pyrrolidin-1-yl) prop-2-yn-1-yl)phenol

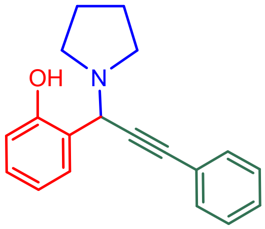

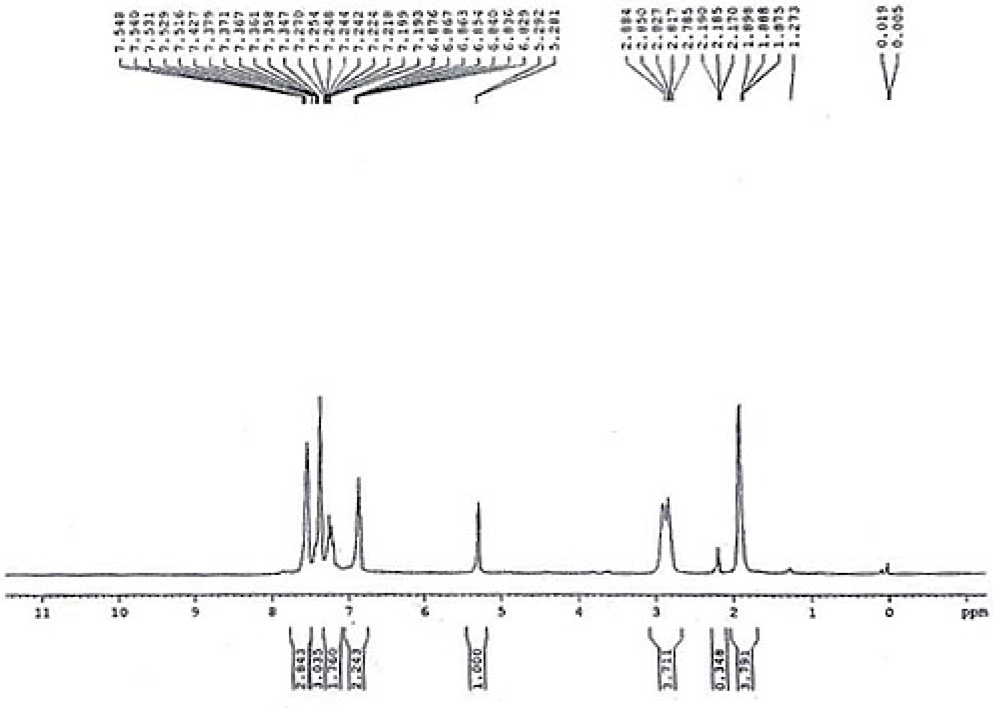

^{1}H NMR

2-(3-phenyl-1-(pyrrolidin-1-yl) prop-2-yn-1-yl)phenol

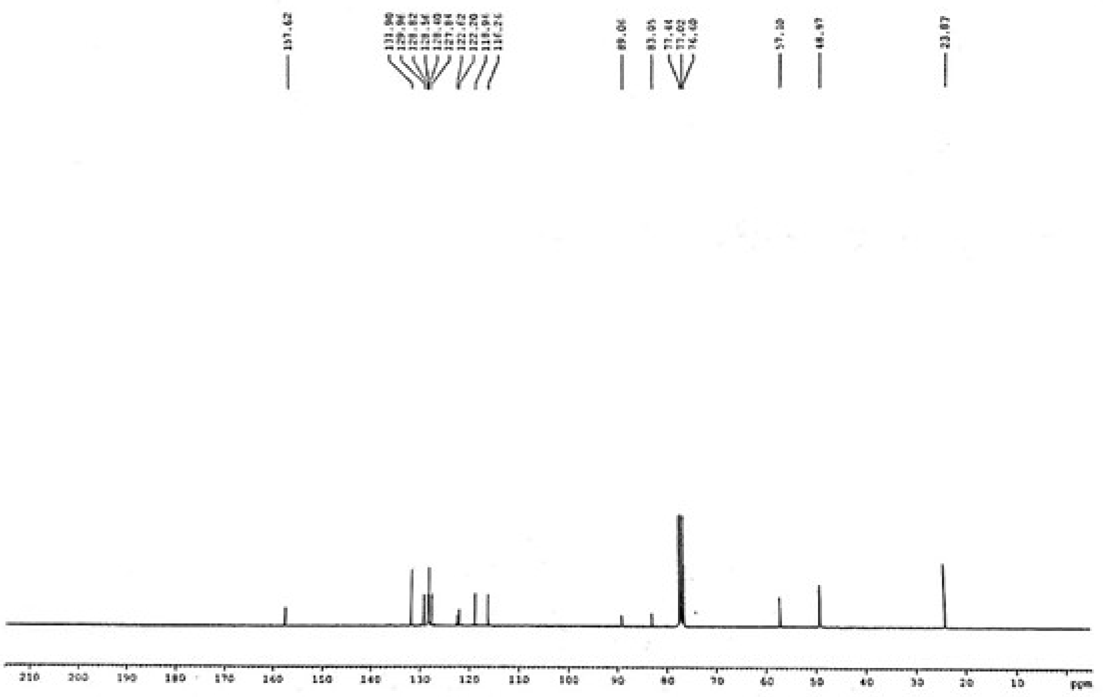

^{13}C NMR

2-(3-phenyl-1-(pyrrolidin-1-yl) prop-2-yn-1-yl)phenol

13-(3-phenyl-1-(thiophen-2-yl) prop-2-yn-1-yl)morpholine

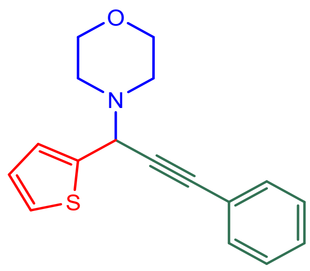

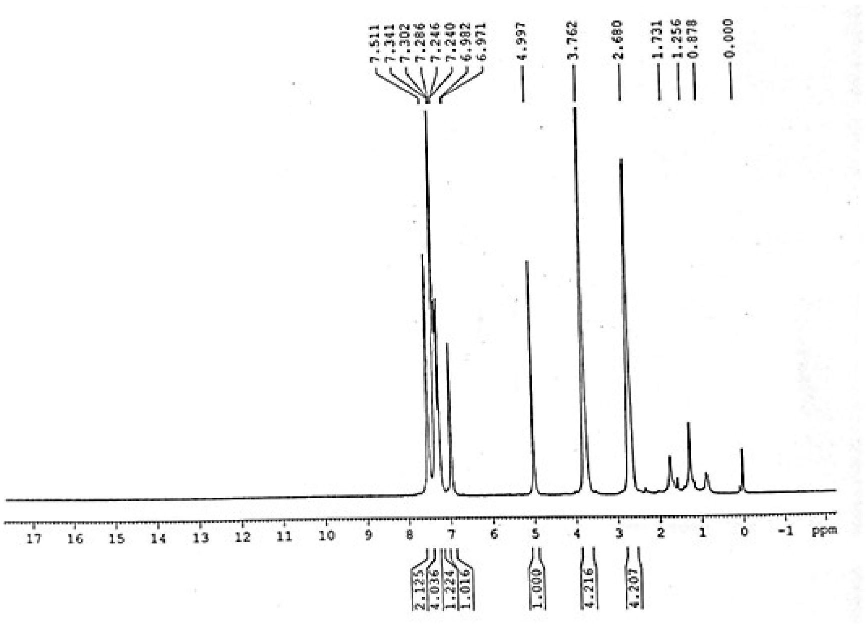

^{1}H NMR

4-(3-phenyl-1-(thiophen-2-yl) prop-2-yn-1-yl)morpholine

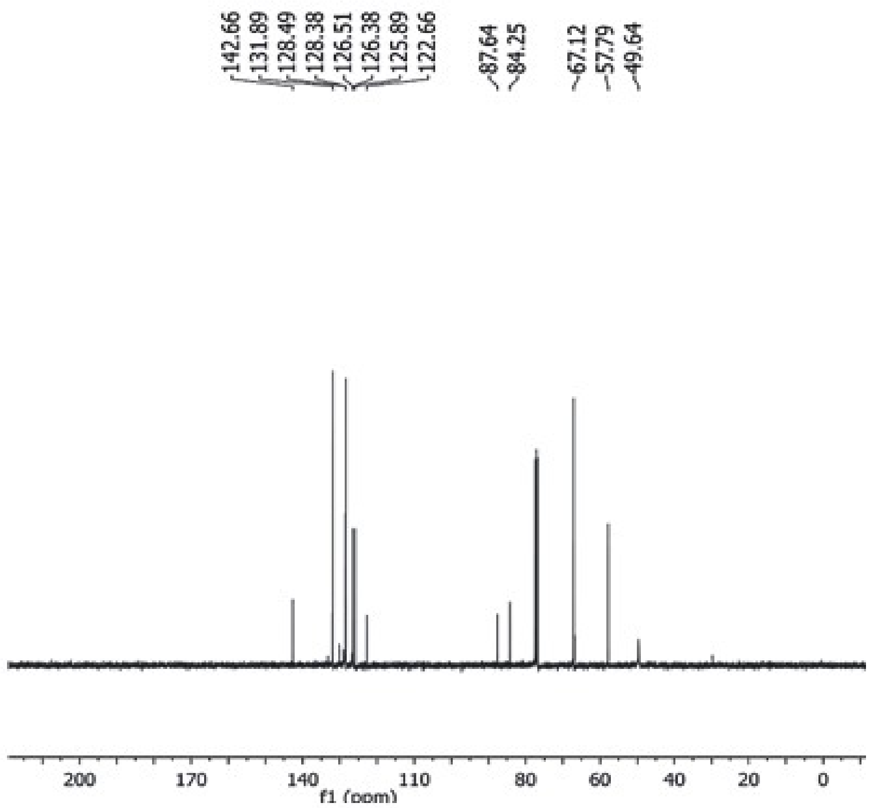

^{13}C NMR

4-(3-phenyl-1-(thiophen-2-yl) prop-2-yn-1-yl)morpholine

14-(1-(2-chlorophenyl)-3-phenylprop-2-yn-1-yl) morpholine

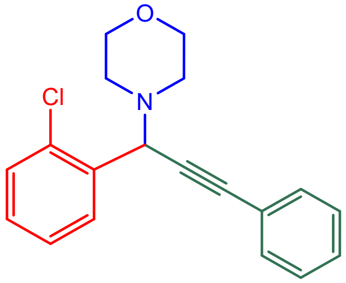

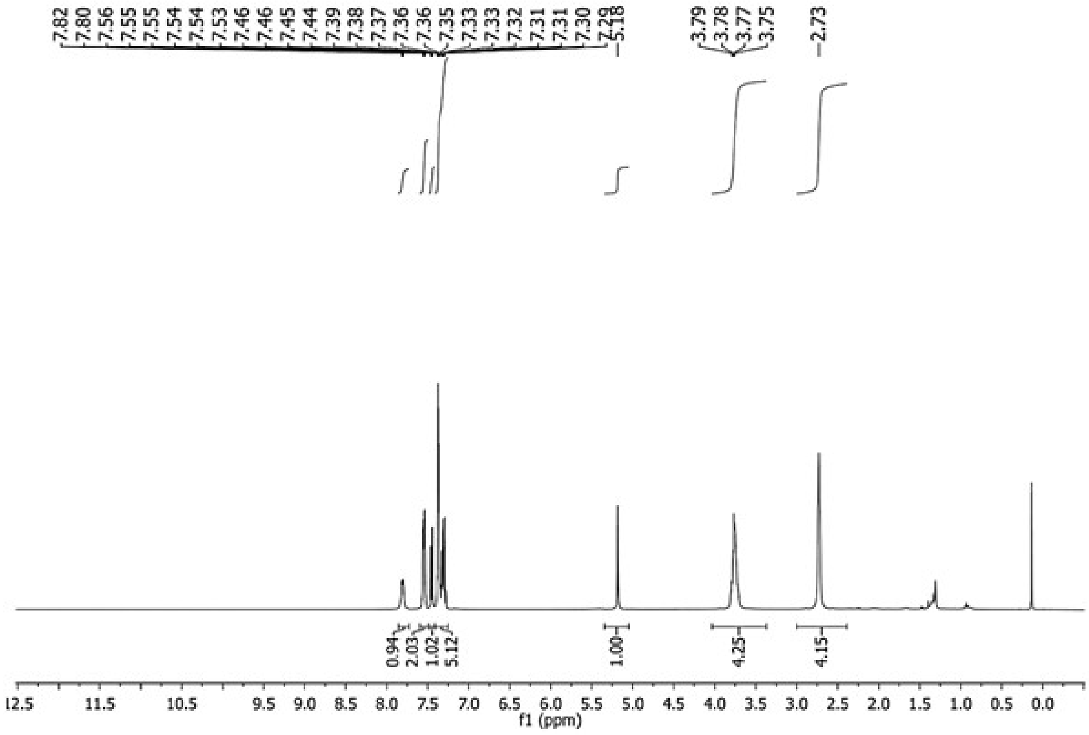

^{1}H NMR

4-(1-(2-chlorophenyl)-3-phenylprop-2-yn-1-yl) morpholine

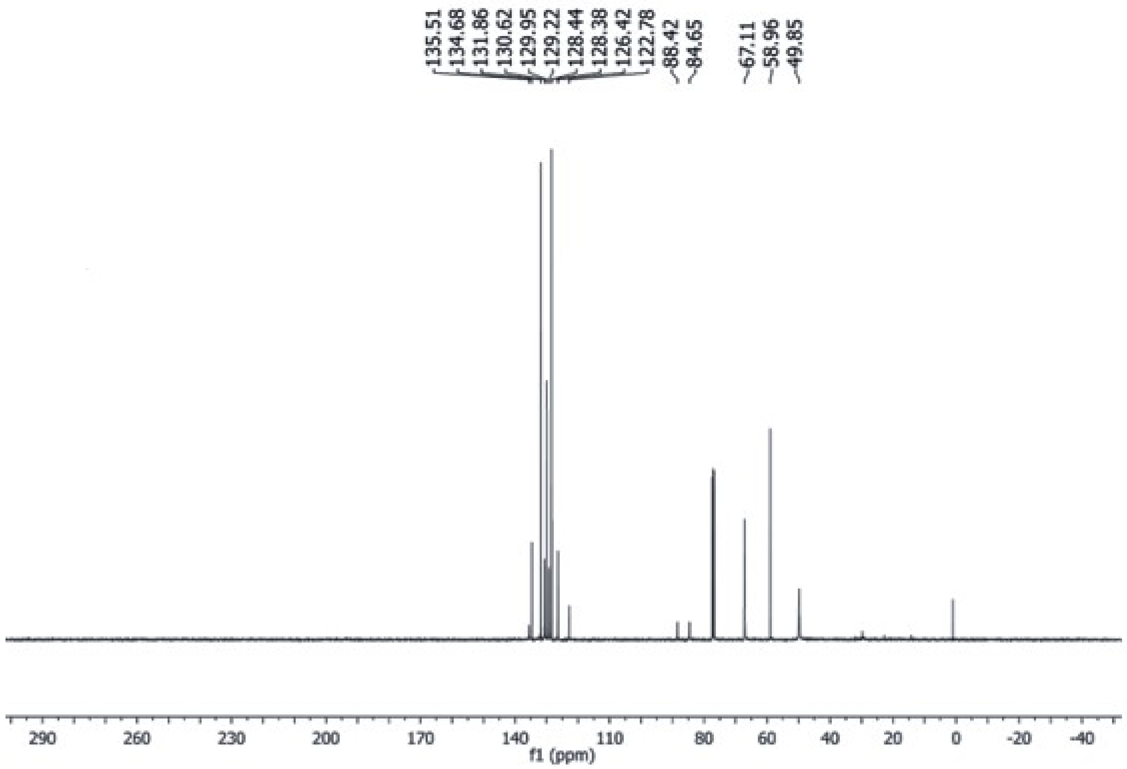

[1]C NMR

4-(1-(2-chlorophenyl)-3-phenylprop-2-yn-1-yl) morpholine

15-(3-phenyl-1-(thiophen-3-yl)prop-2-yn-1-yl)piperidine

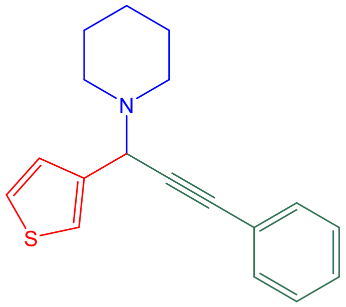

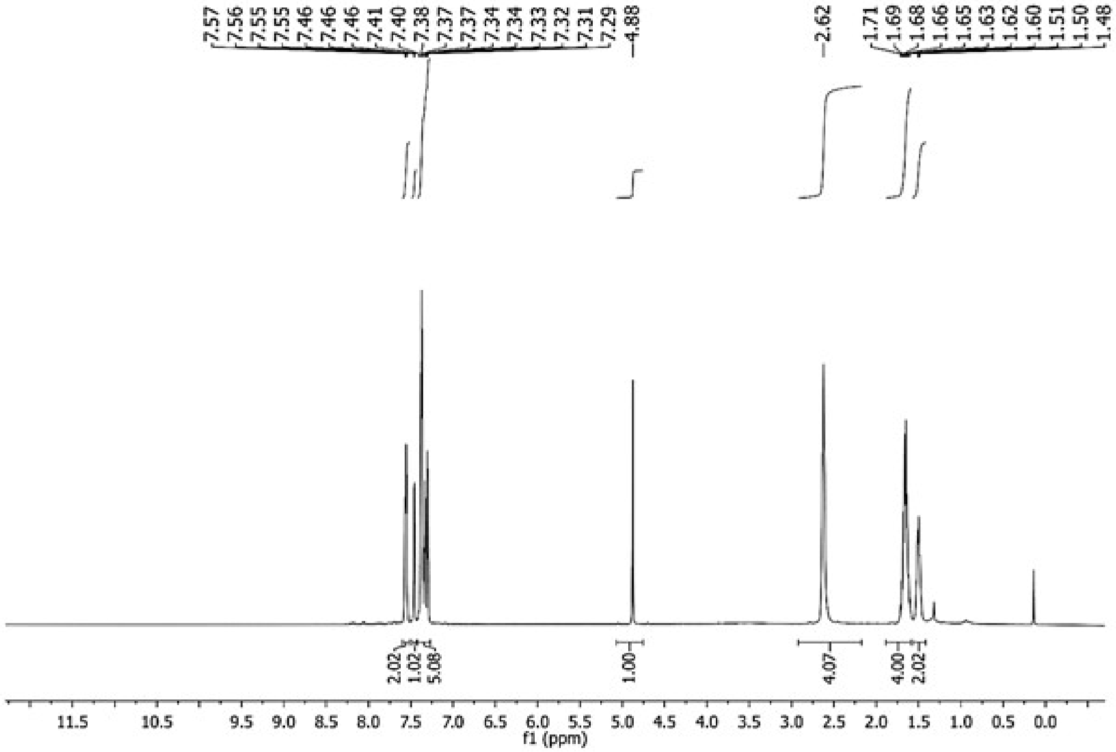

^{1}H NMR

1-(3-phenyl-1-(thiophen-3-yl) prop-2-yn-1-yl)piperidine

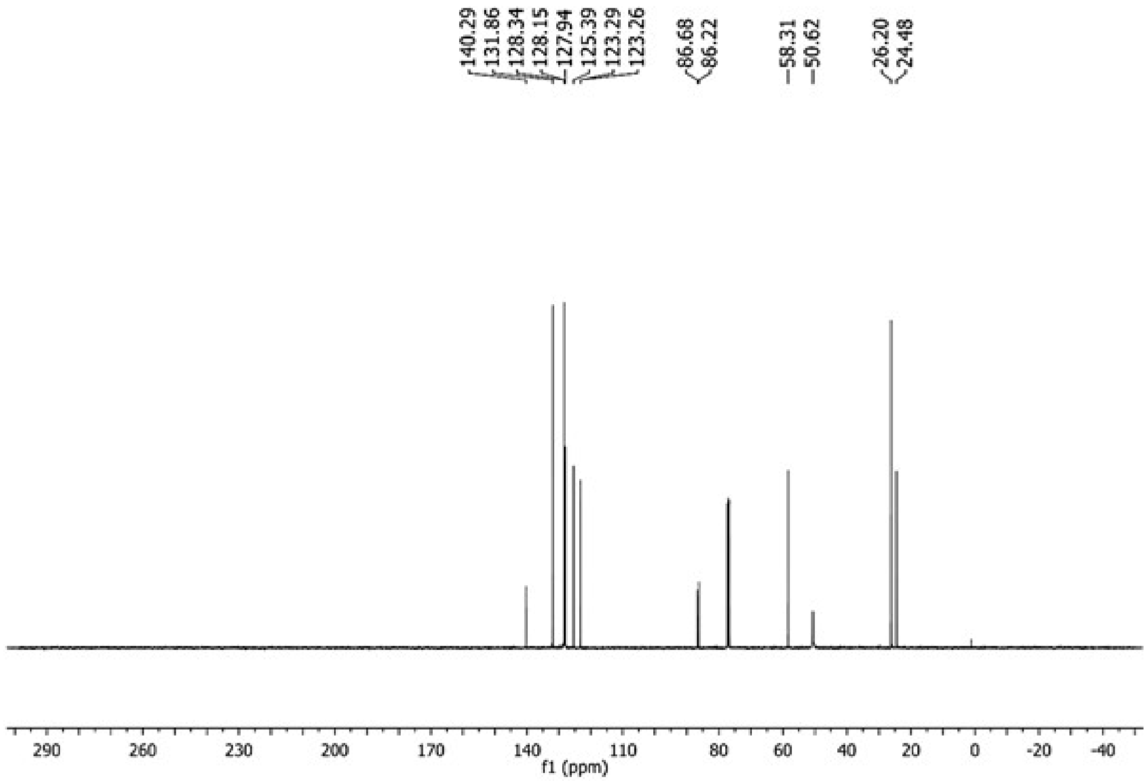

^{13}C NMR

1-(3-phenyl-1-(thiophen-3-yl) prop-2-yn-1-yl) piperidine

References

1 N. V. Tzouras, S. P. Neofotistos, and G. C. Vougioukalakis, *ACS Omega.*, 2019, **4**, 10279 – 10292.

2 A. Neshat, M. Gholinejad, M. Afrasi, P. Mastrorilli, S. Todisco, S, Gilanchi and F. Osanlou, *Appl. Organomet. Chem.*, 2021, **35**, e6180.

3 X. Zhian, X. Jinxi and L. Yiqun, *Appl. Organomet. Chem.*, 2021, **35**, e6349.

4 J.-Y. Zhang, X. Huang, Q.-Y. Shen, J.-Y. Wang and G.-H. Song, *Chin. Chem. Lett*, 2018, 29, 197-200.

5 M. Gholinejad, F. Saadati, S. Sheybanizadeh and B. Pullithadathil, *RSC Adv*, 2016, **6**, 4983-4991
